# mTOR pathway gene mutations predict response to immune checkpoint inhibitors in multiple cancers

**DOI:** 10.1186/s12967-022-03436-1

**Published:** 2022-05-31

**Authors:** Lei Cheng, Yanan Wang, Lixin Qiu, Yuanyuan Chang, Haijiao Lu, Chenchen Liu, Bo Zhang, Yan Zhou, Hao Bai, Liwen Xiong, Hua Zhong, Wei Nie, Baohui Han

**Affiliations:** 1 Department of Pulmonary Medicine, Shanghai Chest Hospital, Shanghai Jiaotong University, Shanghai, 20030 China; 2 Department of Medical Oncology, Fudan University Shanghai Cancer Center, Shanghai, 200032 China; 3 Department of Gastric Surgery, Fudan University Shanghai Cancer Center, Shanghai, 200032 China

**Keywords:** mTOR pathway, Cancer, ICI, TMB, Prognosis

## Abstract

**Background:**

mTOR pathway is known to promote cancer malignancy and influence cancer immunity but is unknown for its role in immune checkpoint inhibitors (ICI) therapy.

**Methods:**

Using Memorial Sloan-Kettering Cancer Center dataset (MSKCC), we extracted mTOR pathway gene mutations for stepwise Cox regression in 1661 cancer patients received ICI. We associated the mutation of the gene signature resulted from the stepwise Cox regression with the 1661 patients’ survival. Other 553 ICI-treated patients were collected from 6 cohorts for validation. We also performed this survival association in patients without ICI treatment from MSKCC as discovery (n = 2244) and The Cancer Genome Atlas (TCGA) as validation (n = 763). Pathway enrichment analysis were performed using transcriptome profiles from TCGA and IMvigor210 trial to investigate the potential mechanism.

**Results:**

We identified 8 genes involved in mTOR pathway, including *FGFR2*, *PIK3C3*, *FGFR4*, *FGFR1*, *FGF3*, *AKT1*, *mTOR*, and *RPTOR*, resulted from stepwise Cox regression in discovery (n = 1661). In both discovery (n = 1661) and validation (n = 553), the mutation of the 8-gene signature was associated with better survival of the patients treated with ICI, which was independent of tumor mutation burden (TMB) and mainly attributed to the missense mutations. This survival association was not observed in patients without ICI therapy. Intriguingly, the mutation of the 8-gene signature was associated with increased TMB and PD1/PD-L1 expression. Immunologically, pathways involved in anti-tumor immune response were enriched in presence of this mutational signature in mTOR pathway, leading to increased infiltration of immune effector cells (e.g., CD8 + T cells, NK cells, and M1 macrophages), but decreased infiltration of immune inhibitory M2 macrophages.

**Conclusions:**

These results suggested that mTOR pathway gene mutations were predictive of better survival upon ICI treatment in multiple cancers, likely by its association with enhanced anti-tumor immunity. Larger studies are warranted to validate our findings.

**Supplementary Information:**

The online version contains supplementary material available at 10.1186/s12967-022-03436-1.

## Introduction

Immune escape of cancer cells driven by immune checkpoints, including PD-1 and CTLA4, yielded hot-spots to be targeted in the recent years [1, 2]. The treatment of immune checkpoint inhibitors (ICI) prolonged survival for cancer patients [3, 4] and had been adopted in neoadjuvant therapy [5] and frontline therapy [6] of advanced cancers. However, the prediction of the ICI response remains challenging [7], because a considerable number of patients cannot obtain long-term clinical benefit from the agent in presence of well recognized predictors, including microsatellite instability (MSI) [8], high PD-L1 expression [9], and high tumor mutation burden (TMB) [10]. In the clinical practice, the survival upon ICI treatment also seemed different among patients with similar level of above biomarkers, highlighting the need for innovative biomarkers to guide ICI treatment decision.

mTOR pathway was well recognized as simulators for cancer malignancy in multiple cancers. Recently, it was also known as key modulator for cancer immunity by its function in cell metabolism [11–13]. Deletion of mTOR blunted immune cell function [13] and down-regulated the PD-L1 expression in cancers [14]. It was reported that the functional loss of mTOR caused abolishment of response to cytokines, followed by silence of transcription factors specific for CD4 + T helper linage differentiation [15, 16]. Unlike these CD4 + linage, mTOR pathway activation might antagonize T regulatory cells differentiation, likely due to the stimulation of anabolic glycolysis metabolism but not fat acid production needed for T regulatory cells generation [3, 17]. Of notice, the activation of mTOR complex1 (mTORC1) promoted a series of transcription factors, followed by increased production of enzymes prerequisite for lipid synthesis, glycolysis, and glutaminolysis, leading to metabolism switch from naive T cells to effector T cells [18, 19]. Collectively, these previous observations advance our understanding of the role mTOR pathway may play in metabolism reprogramming that is essential for generation of immune effector cells in cancer microenvironment.

Because of the relationship between mTOR pathway and cancer immunity, we hypothesized that genetic mutations in mTOR pathway may affect the cancer immunity microenvironment and predict the response to ICI treatment for cancer patients. To test this hypothesis, we performed a two-stage survival association study and identified an 8-gene signature in mTOR pathway whose mutation was associated with better survival in cancer patients received ICI treatment. Mutation of this 8-gene signature was associated with increased genetic alterations in DNA repair genes, which was a potential reason underlying its association with increased TMB. The TMB state did not alter the trend towards better response to ICI treatment in presence of the mutation in the mTOR pathway signature, highlighting its potential use in clinical practice independent of TMB. Importantly, this pathway-derived mutation caused enrichment in metabolism and anti-tumor immune pathways, leading to increased infiltration of immune effector cells in cancers. Together, mTOR pathway mutations in tumors may result in immunologically “hot” microenvironment sensitive to ICI treatment.

## Materials and methods

**Fig. 1:**
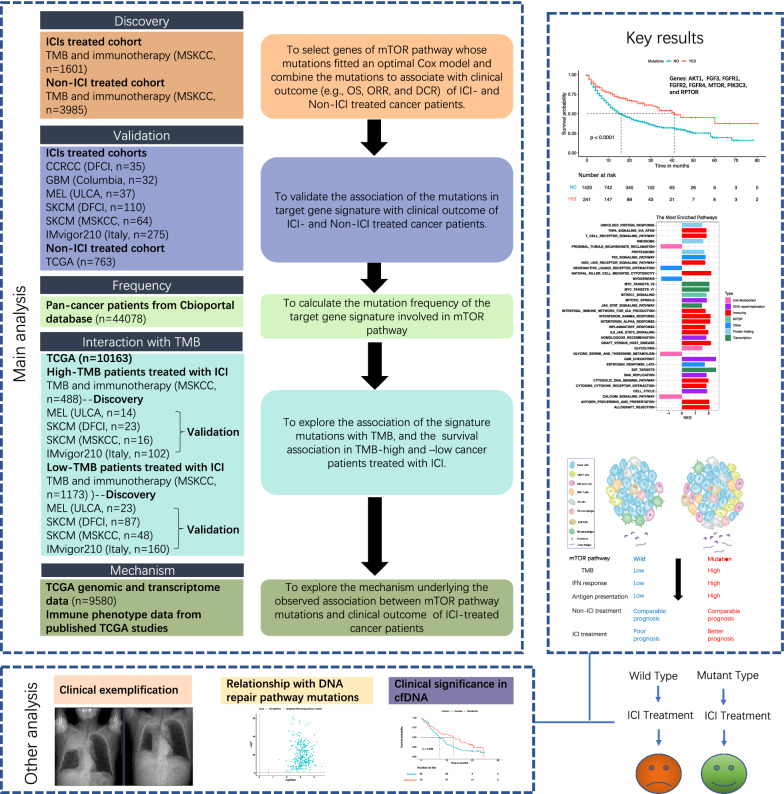
The workflow and summary of the present study

We presented the workflow and summary of the present study in Fig. 1.

### Genes identification in mTOR pathway

We searched for PathCards database (https://pathcards.genecards.org) and identified genes involved in mTOR pathway. We compared the MSK-IMPACT, PGDx elio Tissue Complete, and Foundation One, the three widely used next generation sequencing (NGS) panel in clinical trial of TMB, for the mTOR pathway genes involved. We next scanned the mutation information in our discovery dataset of Memorial Sloan-Kettering Cancer Center (MSKCC) study and identified genes with available mutation information for further survival analysis.

### Patients and two-stage survival analysis

We included patients who received at least one dose of ICI treatment for two-stage survival analysis. The ICI treatment including anti-PD1 (e.g., nivolumab and pembrolizumab), anti-PDL1 (e.g., atezolizumab, avelumab, and durvalumab) and anti-CTLA4 (e.g., ipilimumab and tremelimumab) agent. In the discovery, we included 1661 patients from a MSKCC cohort that investigated the association of TMB and ICI treatment [10]. We incorporated the 23 mTOR pathway genes with available mutation information into stepwise Cox proportional hazards regression model. Then, we generated the optimal fitted Cox model and selected the variables/genes iterated out. We integrated the variables/genes as a whole signature to explore the survival association in the comparison of mutant- versus wild-type cancer patients treated with ICI. In the validation, we included other 553 patients treated with ICI from other 6 independent published studies for survival association [20–25]. To exclude the possibility that mTOR pathway mutations was a prognostic marker regardless of the ICI treatment, we also associated the mutation states with survival of non-ICI treated patients with 2244 patients from the MSKCC study as discovery and 763 advanced cancer patients from TCGA as validation. All survival analysis were visualized by Kaplan–Meier curve with log-rank test. To quantify the death risk, we also perform multivariate Cox regression for survival analysis with adjustment of age, gender, TMB and treatment. We adopted the restricted mean survival time (RMST) method for multivariate survival comparisons in the case of unmet requirements for Cox assumptions. Hazard ratio (HR) and life expectancy ratio (LER) were used to express the adjusted results for Cox and RMST model, respectively. Because the data for objective response rate (ORR) and durable clinical benefit (DCB) was limited, we also combined the two-stage patients available for these data and compare the ORR and DCB based on the mutation states of the signature using chi-square test.

### Mutation frequency and distribution across cancer types

We extracted 48,834 cancer patients from 188 non-redundant studies deposited in cbioportal database, and further identified 44,078 patients with available mutation information in 8-gene signature in mTOR pathway, including *FGFR2*, *PIK3C3*, *FGFR4*, *FGFR1*, *FGF3*, *AKT1*, *mTOR*, and *RPTOR*. We calculated the mutation frequency of these 8 genes as well as their integrated mutation frequency across multiple cancer types. The MAF files of TCGA were also used to plot the mutation exclusion pattern among them.

### Mutation types and survival

We performed subgroup analysis according to the mutation types to explore its effect on patients’ survival upon immune checkpoint blockade. Because of the limited sample size in some rare types of mutations, patients were divided into those with missense mutation and others without this type of mutation. Then Kaplan–Meier plot with log-rank test, along with multivariate analysis (e.g., Cox regression for discovery and RMST analysis for validation) were used for survival comparison.

### Interaction with TMB and survival

We associated the mutation states of the 8-gene signature with TMB of 5646 cancer patients from MSKCC cohort and validated this association in TCGA including 10,163 patients. Wilcoxon rank-sum test was used to compare the means of TMB between two groups because of the abnormal distribution of the data. We also tested the survival association of the mutation states in low- (< 10 mut/Mb) and high-TMB (> 10 mut/Mb) patients treated with ICI in MSKCC cohort. In the validation of independent published studies for survival interaction with TMB, a cutoff of 10mut/Mb was also used in the next-generation sequencing study for TMB classification, and the upper quartile of TMB was used for classification in whole-exon sequencing studies. Because decreased sample size in subgroup division that may introduce statistical bias, we also pooled the results in discovery and validation by meta-analysis with random effects model.

### Mutations and the activation status of mTOR pathway

As we know, the activation or inactivation of mTOR pathway influence its target genes. To explore whether the mutations may affect the activation status of mTOR pathway, we associated the 8-gene signature mutation in mTOR pathway with mRNA expression of the well recognized target genes using Wilcoxon rank-sum test.

### Immunological phenotype and mechanism

To explain the relationship between 8-gene signature mutation in mTOR pathway and increased TMB, we asked whether there is an association between the signature mutation with genetic mutations involved in DNA damage response (DDR) pathway. Then, we extracted the transcriptome data to calculate immune phenotype (e.g., immune cells inflammation, antigen presentation, and neoantigen production) of cancer patients according to published TCGA studies [26, 27]. With Wilcoxon rank-sum test, we explored the impact of the signature mutation on immune phenotype using TCGA pan-cancer database. Specially, we listed the genes involved in cancer immune casecade, including antigen presentation, costimulatory/coinhibitory signals, related ligand and receptor, and cell adhesion. We computed the fold change in these genes in the comparison of mutant- versus wild-type patients and visualized it in one heatmap. We also explored the impact of the 8-gene signature mutation on expression of immune checkpoint and chemokine genes using Wilcoxon rank-sum test. Finally, gene set enrichment analysis (GSEA) was carried out to investigate the potential mechanism underlying the observation of immune phenotype in the comparison of mutant- versus wild-type patients. We further validated the GSEA results from TCGA by 275 advanced cancer patients in IMvigor210 clinical trial.

### Clinical case report

We reported one case with advanced lung squamous cell carcinoma in our center with AKT1 mutation, who was received two cycle treatment of 200 mg pembrolizumab upon initial diagnosis. The patient has signed a written informed consent to denote related clinical data for the purpose of scientific research. We got the computed tomography images before and after the pembrolizumab treatment, in order to visualize the treatment response.

### Mutations in circulating free DNA (cfDNA) and ICI treatment

By extraction of sequencing data from blood TMB study [28], we investigated whether there was a survival association of the cfDNA mutations in mTOR pathway with ICI treatment outcome. We also explored the clinical outcome in the comparison of docetaxel versus atezolizumab treatment in mutant-type patients.

### Statistical tools

We performed all survival analysis and genetic analysis in the present study by R version 4.1.0, with two-side *P* < 0.05 deemed as statistically significant.

## Results

### mTOR pathway genes, patients, and two-stage survival analysis

**Fig. 2:**
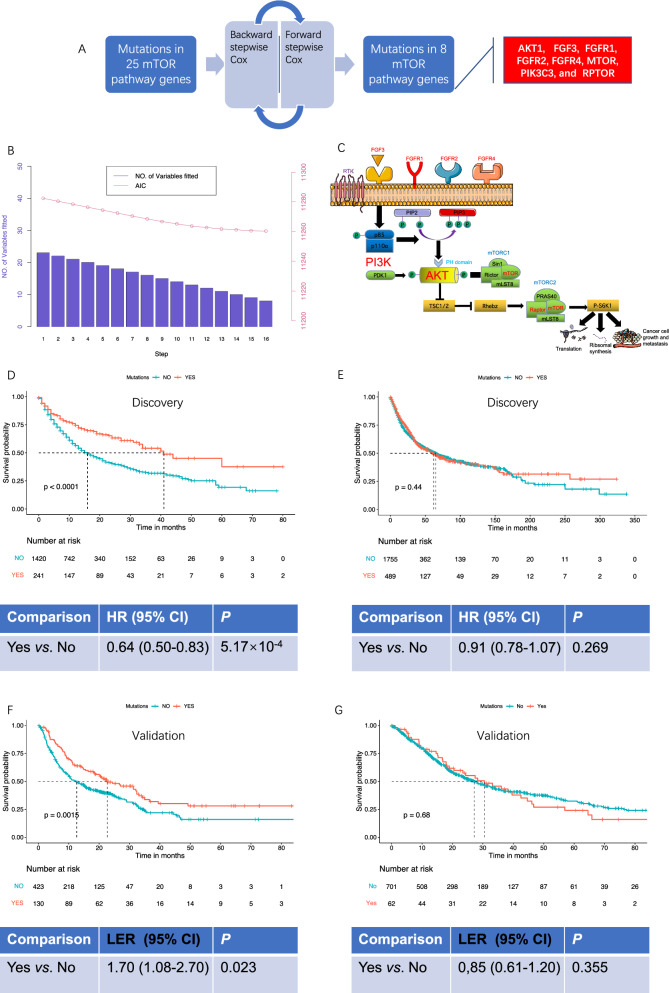
Identification of mTOR pathway genes and two-stage survival analysis in cancer patients with ICI treatment. In discovery stage of 1661 ICI-treated cancer patients from TMB and immunotherapy study by MSKCC, we performed stepwise Cox regression with both direction of backward and forward using 23 mTOR pathway genes, which further yielded 8 genes upon iteration, including *FGFR2*, *PIK3C3*, *FGFR4*, *FGFR1*, *FGF3*, *AKT1*, *mTOR*, and *RPTOR* (**A**). The AIC and variables left for each step during stepwise Cox regression (**B**). The biological role of the 8 genes, which were marked red, in mTOR pathway (**C**). Mutation states of the 8-gene signature for the 1661 ICI treated patients were used for integrated survival comparison by Kaplan–Meier curves and multivariate Cox regression with adjustment for age, gender, treatment and TMB (**D**). The survival association was also tested in 2244 patients without ICI treatment (**E**). Using RMST analysis, the survival association were validated in 553 ICI-treated patients from other 6 independent cohorts and 763 patients without ICI treatment from TCGA (**F**, **G**)

We conducted a two-stage analysis (Additional file 2: Table S1) to explore and validate the association of mutations in mTOR pathway genes with survival of patients treated with ICI. The patients’ characteristics were presented in Additional file 2: Tables S2, S3. We found that compared with the PGDx elio Tissue Complete and Foundation One NGS panel, the MSK-IMPACT panel had the most genes involved in mTOR pathway. We identified 23 mTOR pathway genes (Additional file 2: Table S4) with available mutation information in 1661 patients from MSKCC study of TMB and immunology. By incorporating mutations in these genes in stepwise Cox regression, we further identified 8 genes, including *FGFR2*, *PIK3C3*, *FGFR4*, *FGFR1*, *FGF3*, *AKT1*, *mTOR*, and *RPTOR*, that fitted an optimal survival model for patients treated with ICI (Fig. 2A). The stepwise Cox analysis considered both forward and backward direction and resulted in decreased akaike information criterion (AIC) and variables as the increase in iteration (Fig. 2B). Of note, the 8 genes were found to be located at the key position of the mTOR pathway, including the ligand and receptor, and genes involved in midstream phosphorylase kinase (Fig. 2C). We next asked the survival association of mutation states for this 8-gene signature by integrated analysis. We observed prolonged survival by mutations of this 8-gene signature in mTOR pathway, with death risk decreased by 36% (HR = 0.64, 95%CI = 0.50–0.83, *P* = 5.17 × 10^–4^) upon multivariate adjustment for age, sex, TMB, and treatment (Fig. 2D). Because of unmet assumptions for Cox model, we used RMST method and successfully validated this survival association in other 553 patients from 6 published trials who received ICI (LER = 1.70, 95%CI = 1.08–2.70, *P* = 0.023, Fig. 2F). In 2244 patients who did not receive ICI in the MSKCC study, we did not find any association between the mutations and patients’ survival (Fig. 2E). The lack of survival prediction for patients with non-ICI treatment was also validated in 763 advanced cancer patients from TCGA (Fig. 2G), indicating the specific predictive ability of the mutations in mTOR pathway genes for ICI treatment efficacy.

### Mutation frequency, types, and distribution across cancer types

**Fig. 3:**
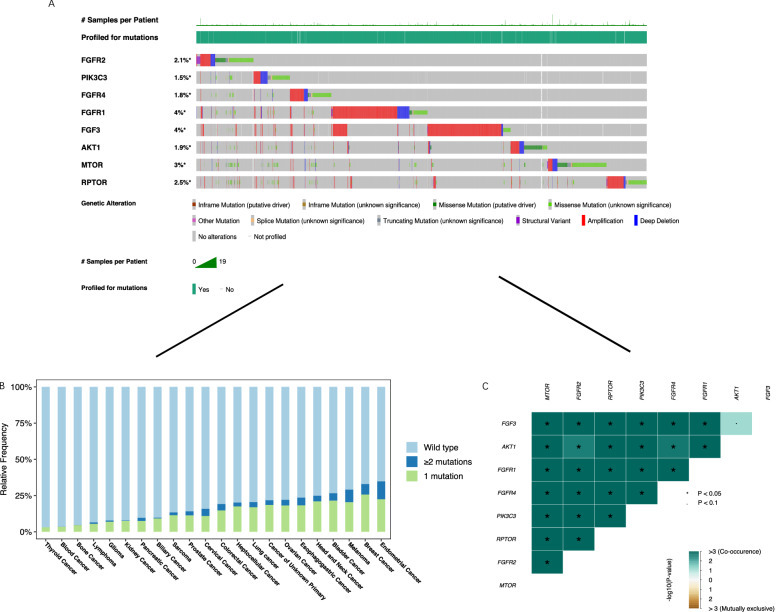
Mutation frequency and distribution across cancer types. The mutant frequency for the 8 genes using 44,078 cancer patients from 188 non-redundant cancer studies deposited in cbioportal database (**A**). The integrated mutation frequency for the 8 genes across multiple cancer types (**B**). The mutation exclusive pattern of the 8 genes by analysis of the TCGA MAF files (**C**)

Using 44,078 cancer patients from 188 non-redundant studies deposited in cbioportal database, we found that the mutant frequency for the 8 genes ranged from 1.5 to 3% (Fig. 3A). Endometrial cancer had the leading mutation frequency by integrated analysis of the 8-gene signature in mTOR pathway, followed by breast cancer and melanoma (Fig. 3B). By analysis of MAF files of TCGA pan-cancer mutations, we found that most of them displayed significant co-mutation (Fig. 3C).

### Mutation types and survival

In both discovery (Additional file 1: Fig. S1A, B) and validation (Additional file 1: Fig. S1C, D), we found that the impact of mTOR pathway mutations on better survival of patients with ICI treatment was mainly attributed to the missense mutations, and we did not find any association of other mutations with patients’ survival. We also visualized the multivariate results in forest plot, in order to present the impact of overall, missense and other mutations, respectively, on patients’ survival upon ICI treatment (Additional file 1: Fig. S1E, F).

### Interaction with TMB and survival

**Fig. 4:**
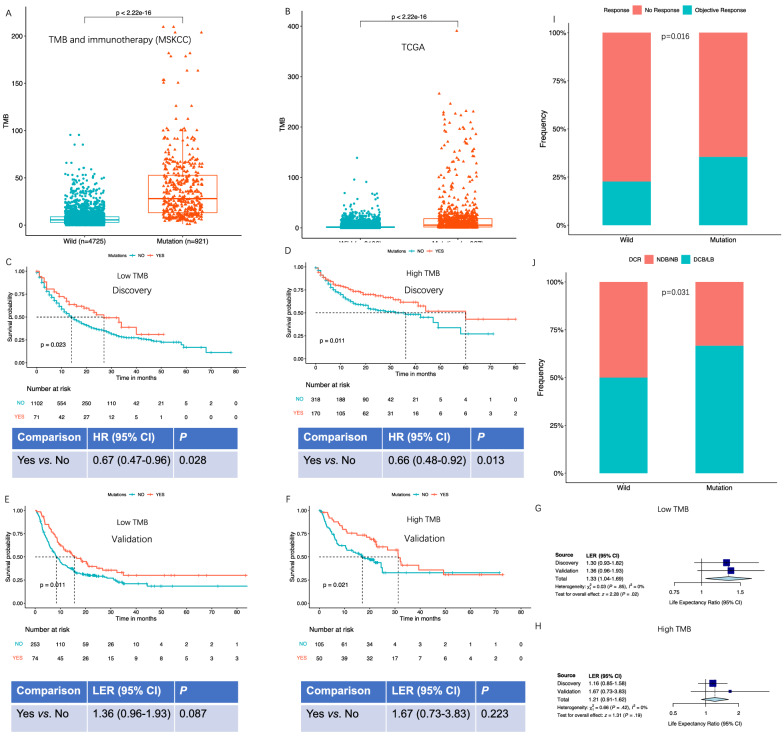
Interaction with TMB and survival. The association of the mutations in the 8-gene signature with TMB in 5646 patients from MSKCC study (**A**) and the validation in 10,163 patients from TCGA database (**B**). In discovery stage of 1661 ICI-treated patients, we showed the survival comparison of mutant-type versus wild-type patients in presence of low TMB (n = 1173, **C**) and high TMB (n = 488, **D**) by Kapan-Meier curves and multivariate Cox regression. The survival interaction with TMB were further validated in validation stage of 327 low-TMB (**E**) and 155 high-TMB patients (**F**) by Kapan-Meier curves and multivariate RMST analysis. To alleviate the limited statistical power induced by subgroup stratification, we pooled the adjusted RMST results of the two stages using meta-analysis method in low-TMB patients (**G**) and high-TMB patients (**H**). We also combined the available samples in the two stages to associate the mutation states of the 8-gene signature with treatment response rate (**I**) and DCB rate (**J**)

Mutations in the 8-gene signature was found to be associated with increased TMB in 5646 patients from the MSKCC cohort (Fig. 4A), which was further demonstrated in 10,163 patients from TCGA database (Fig. 4B). To explain the potential reason, we questioned whether there was a correlation between the mutations in mTOR pathway and DDR pathway. As a result, in both MSKCC and TCGA cohort, we found that the mutation frequency of most genes involved in DDR pathway were up-regulated in presence of mutations in the 8-gene signature in mTOR pathway (Additional file 1: Fig. S2). Specially, these up-regulations were involved in DNA double-strand breaks repair (DSBR), nucleotide excision repair (NER), and base excision repair (BER), including ATM, ERCC3 and 4, and BRCA1 and 2 genes, indicating that the abnormal DNA repair might be the inducement to the increased TMB in the patients with mutant-type signature in mTOR pathway (Additional file 2: Tables S5, S6). In MSKCC study, we found that TMB classification did not influence the trend towards better survival for mutant-type patients with adjustment for age, gender, TMB and treatment (HR = 0.67, 95%CI = 0.47–0.96, *P* = 0.028 for low-TMB patients, Fig. 4C; and HR = 0.66, 95%CI = 0.48–0.92, *P* = 0.013 for high-TMB patients, Fig. 4D). Using multivariate RMST analysis, we observed similar trend with borderline significance in the validation (Fig. 4E, F). We also calculated adjusted RMST results in discovery for polling with validation using meta-analysis method. Polling results demonstrated a better survival in presence of mTOR pathway mutations in low-TMB patients (Fig. 4G). In high-TMB patients, the results achieved a borderline trend similar to that in discovery (Fig. 4H). Because there are limited patients with available treatment response data, we also combined the samples in the two stages to extract the response data and found that mutant-type patients had higher treatment response rate as well as DCB rate compared with wild-type patients (Fig. 4I, J).

### Mutations and the activation status of mTOR pathway

The common target genes of mTOR pathway were shown in Additional file 1: Fig. S3A. We observed up-regulation in mRNA expression of EIF4E (EIF4EBP1), AKT1, HIF-1a (mRNA transcription effector of mTOR pathway), S6K (RPS6KA1 and RPS6KB1), but down-regulation in PKC (PRKCA), SGK1, TFEB and ULK1 by the mTOR pathway mutations (Additional file 1: Fig. S3B). Although most have direction towards the activation of mTOR pathway, the deep regulation in protein function, phosphorylation modification for example, are still unclear.

### Immunological phenotype and mechanism

**Fig. 5:**
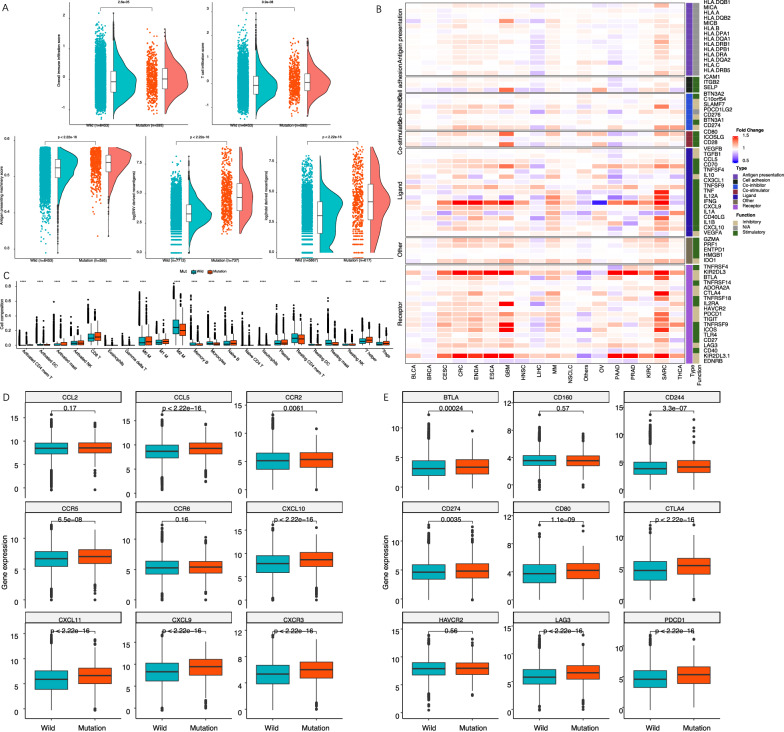
mTOR pathway mutations and immunological phenotype. The association of mutations in the 8-gene signature involved in mTOR pathway with overall immune cells infiltration, T cells infiltration, antigen presentation, and neoantigen production (**A**). A heatmap showed the fold changes of mRNA expression involved in different immune phenotype in the comparison of mutant-type versus wild-type patients (**B**). Specially, we showed the association of the mTOR pathway mutations with expression of chemokines (**D**) and immune checkpoints genes including PD1 and PDL1 (also termed CD274) (**E**) that were closely associated with ICI treatment efficacy. We also observed the change of immune cells infiltration induced by mTOR pathway mutations (**C**); **, < 0.01, ***, < 0.001

**Fig. 6:**
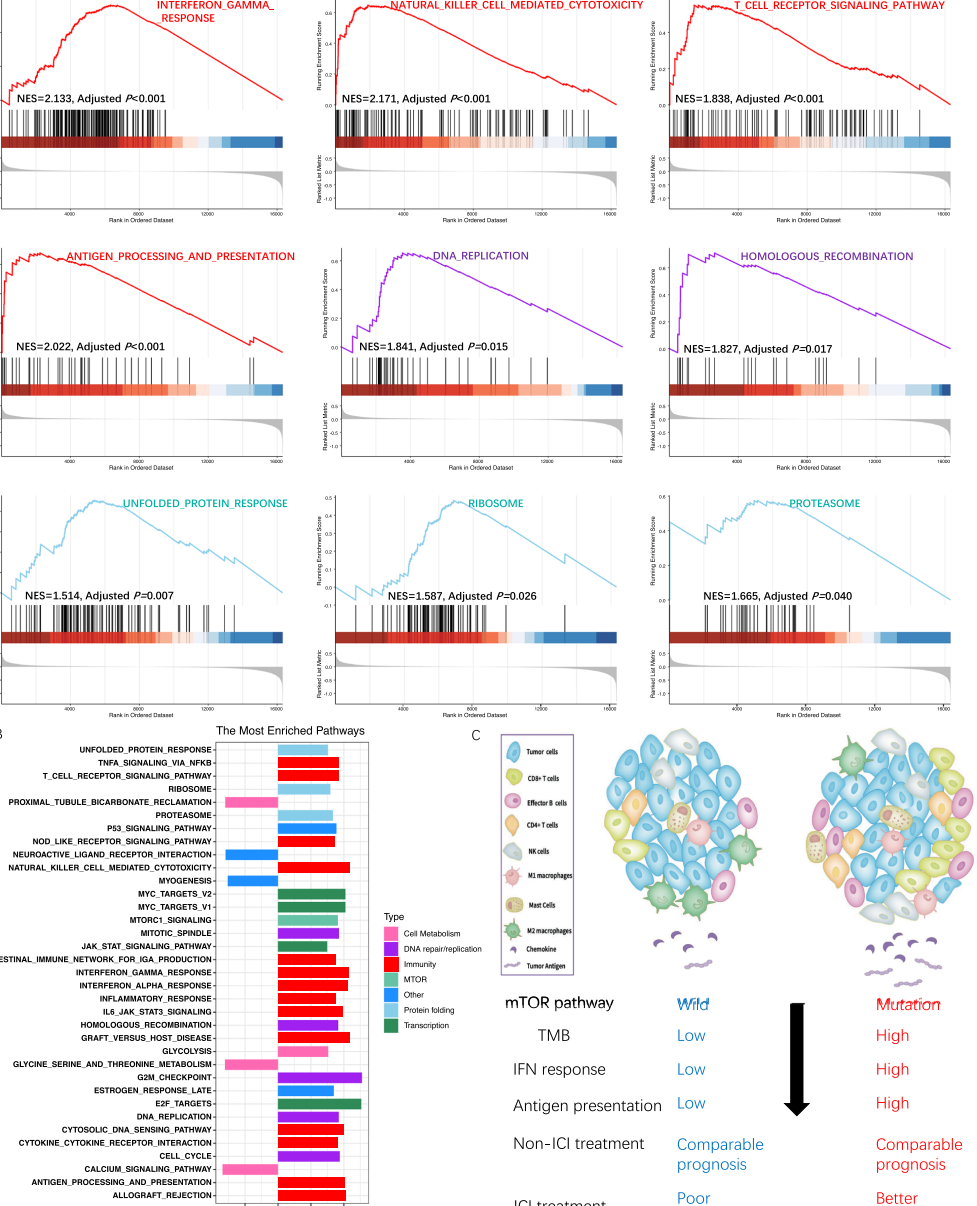
mTOR pathway mutations and pathway enrichment analysis. Pathway enrichment analysis from TCGA database using GSEA method, in the comparison of mutant-type versus wild-type patients for the 8-gene signature involved in mTOR pathway. We showed representative pathway enrichment involved in immunity, DNA repair, and protein folding (**A**). All pathways enriched was summarized in one figure (**B**). Our association analysis of survival, immune phenotype, and potential mechanisms demonstrated the switch from immunologically “cold” to “hot” microenvironment in tumors in presence of the 8-gene signature involved in mTOR pathway (**C**)

Using transcriptome data extracted from TCGA, we found that mutations in the 8-gene signature in mTOR pathway induced higher overall immune cells infiltration, T cells infiltration, antigen presentation, and neoantigen production (Fig. 5A). Specially, we observed the increased infiltration of CD8 + T cells, activated B (plasma) cells, activated mast cells, activated CD4 + memory T cells, M1 macrophages, but decreased M2 macrophages (Fig. 5C). Further TCGA analysis also resulted in the tendency towards up-regulation in mutant-type patients for the mRNA expression involved in different immune phenotype (Fig. 5B), especially for chemokines (Fig. 5D) and immune checkpoints (Fig. 5E), including PD1 and PDL1 (also termed CD274, Fig. 5E) that were closely associated with immune cells recruitment and ICI treatment efficacy. To reveal the potential mechanism underlying our observation in TCGA, we performed pathway enrichment analysis using GSEA method. There was an obvious immune pathway enrichment, such as T cell receptor signaling, NK cell mediated cytotoxicity, antigen presentation (Fig. 6A, B), and interferon response pathway (Fig. 6B), in the comparison of mutant-type versus wild-type patients. As expected, the mTORC1 and metabolism pathway was also enriched (Fig. 6B), because of the function in metabolism reprogramming by mTOR pathway mutation. DNA repair and protein folding, two pathways that caused production of neoantigen, were also enriched by the signature mutation in mTOR pathway (Fig. 6A, B). We repeated the GSEA analysis in transcriptome data from IMvigor210 clinical trial, and validated the enriched pathways mainly overlapped in immunology (Additional file 1: Fig. S4). The results for GSEA analysis in TCGA and IMvigor210 in detail were presented in Additional file 2: Tables S7 and S8, respectively. Taken together, our observations provided the clues for the association between mTOR pathway mutations and “hot” microenvironment in tumors (Fig. 6C). We finally exemplified an 82-year-old patient with advanced lung squamous cell carcinoma in our center, who had AKT1 mutation in the tumor and good response to pembrolizumab. The tumor has shrunk by approximately two thirds after 2 cycle treatment of 200 mg pembrolizumab (Additional file 1: Fig. S5).

### Mutations in circulating free DNA (cfDNA) and ICI treatment

Blood-based NGS was performed in OAK and POPLAR trial with comparison of ICI treatment versus chemotherapy, which was extracted for the association analysis between the mTOR mutations and ICI treatment response. We identified 853 patients with cfDNA mutation data, of which 424 patients received docetaxel and 429 patients received atezolizumab. Among these patients, 157 had mutations in the 8-gene signature in mTOR pathways, including 85 patients treated with docetaxel and 72 patients treated with atezolizumab. We did not find any significant association of the mutations in mTOR pathway signature with efficacy of atezolizumab treatment (data not shown). However, compared with docetaxel, we found that mutant-type patients had prolonged overall survival (OS) (Additional file 1: Fig. S6A), progression-free survival (PFS) (Additional file 1: Fig. S6B), and increased ORR and DCB (Additional file 1: Fig. S6C, D) in the comparison of atezolizumab versus docetaxel treatment. In the subgroup analysis by TMB, survival remained favorable in mutant-type patients treated with atezolizumab in comparison with docetaxel (Additional file 1: Fig. S7), except for PFS in high-TMB patients that only reached insignificant trend in multivariate RMST analysis (Additional file 1: Fig. S7D).

## Discussion

Genetic mutations [29, 30], transcriptomic signature [31] and DNA methylation [32] were previously reported to be associated with sensitivity and resistance to ICI treatment. In the present study, we have reported that mutations in mTOR pathway, a classical modulator in cancer cell metabolism and cancer malignancy, were predictable for ICI treatment outcome. mTOR pathway induces the metabolism switch needed for immune activation [12]. Indeed, in presence of mTOR pathway mutations, we observed pathway enrichment involved in anti-tumor immunity in tumors, as well as enhanced antigen presentation and increased infiltration in immune effector cells.

There were some previous studies exploring the association between mTOR pathway mutations and ICI treatment. One multi-omics study demonstrated that mTOR and PI3K-AKI axis were involved in gastric cancer immunity, and the related mutations were associated with better survival of patients with anti-PD-1/PD-L1 immunotherapy [33]. In another study, it was reported that PIK3CA gene mutation was associated with immunity in bladder cancer, and its inhibitor increased the sensitivity to PD1 blockade for mutant-type patients [34]. Intriguingly, like our studies, it was previously reported that the AKT-MTOR axis mutation was able to upregulate immune checkpoints [14], including PDL1, a widely used biomarker that sensitize the ICI treatment in a series of studies. In the present study, we develop and validate the role of the mutations in mTOR pathway genes, including *FGFR2*, *PIK3C3*, *FGFR4*, *FGFR1*, *FGF3*, *AKT1*, *mTOR*, and *RPTOR*, in ICI treatment response. Not surprisingly, these 8 genes were all located at the key node of the mTOR pathway and are indispensable for the downstream activation, providing the ration for their association with ICI treatment. Collectively, mTOR pathway mutations are obviously involved in cancer immunity, and affect the sensitivity to ICI treatment.

Of note, we observed that mutations in mTOR pathway genes were positively associated with DDR pathway mutations. We also found the enrichment in DNA repair pathway by mTOR pathway mutation in tumors. In previous studies, mTOR pathway was reportedly to be interacted with DDR, including DNA repair pathway [35, 36]. The link of mTOR pathway with DDR pathway provided another mechanism underlying the predictive ability of mTOR pathway mutation for ICI treatment response, because DNA repair pathways plays a role in TMB accumulation and neoantigen production, thus they were previously demonstrated to be associated with sensitivity of ICI treatment [37]. Another finding in the present study is also rational that mTOR pathway mutation was associated with increased TMB, likely due to its close relationship with DNA repair processing.

Intriguingly, the stratification analysis suggested that the missense mutations contributed mainly to the better survival for patients with ICI treatment. Because missense mutations may render the protein function, this finding is biological reasonable. Indeed, our further analysis also suggested that the mTOR pathway mutations regulated the mRNA expression in the target genes, but we could not draw a clear conclusion for its impact on the activation status of mTOR pathway due to the lack of protein modification results. However, these results provided some new clues for the involvement of mTOR pathway in ICI treatment. Interaction with TMB was another concern of the findings in the present study. High TMB in cancer tissues was an indicator for sensitive response to ICI treatment as reported by recent studies [10, 38]. Although we observed higher TMB in presence of the mutations in mTOR pathway, TMB had minimal impact on the association between mTOR pathway mutations and ICI treatment sensitivity. Therefore, mutations in this 8-gene signature in mTOR pathway may be used in clinical practice, independent of TMB, to predictive the sensitivity of ICI treatment. Not only TMB in tumor tissues, but also the blood TMB also played a role in ICI treatment efficacy [28, 39]. However, unlike tumor mutation, we did not find any association between cfDNA mutations in mTOR pathway and response to ICI treatment. The results suggest that tumor mutation, not the cfDNA mutation in mTOR pathway, mainly contribute to the immune stimuli in tumor micro-environment and promote the ICI treatment sensitivity in turn.

Researches in recent decades has led to rapid advances in the diagnosis and treatment of cancers [40, 41]. mTOR pathway plays multiple roles in cancer biology that may provides insights into cancer therapy, not limited to ICI treatment. Reactive oxygen species (ROS) formation, an important component in cell metabolism, may activate oncogenic pathways, including ERK1/2 and PI3K/AKT pathways that promote the proliferation and migration of cancer cells [42–44]. Therefore, antioxidant therapy is possible to exert anti-cancer efficiency by dampening ROS production [45]. Autophagy links the mTOR pathway to ROS formation. Of note, authophay induced by mTORC1 suppression in cancer cells may result in degradation of damaged mitochondria that promote the ROS formation and oxidative stress, which is also called mitophagy [46, 47]. Moreover, the activation of mTORC1 suppress cell authophagy in response to increased ROS formation [46]. Taken together, antioxidant therapy is expected to be used in cancer treatment, which may result in different anti-cancer efficacy based on the mTOR pathway activation status. Whether mTOR pathway mutations affect the antioxidant therapy in cancers is to be answered in future investigations.

There are some limitations in the present study. First, the retrospective design in data collection may introduce bias in the present study. Second, the subgroup analysis based on TMB decrease the statistical power to some extent. Third, this is a pan-cancer study, but not all types of cancer are included.

In summary, we demonstrated that mutations in an 8-gene signature in mTOR pathway might promote the ICI treatment sensitivity, which was independent of TMB. As a potential mechanism underlying this association, these mutations were likely to have association with enhanced anti-tumor immunity by increased infiltration of immune effector cells. Larger studies are warranted to validate our findings.

## Supplementary Information

**Additional file 1: Figure S1. **Mutation types of mTOR pathway and survival of patients with ICI treatment. The association of the missense mutations and other mutations in mTOR pathway with survival patients who underwent ICI treatment, respectively (A-B). The validation for this association stratified by mutation types (C-D). Visualization in discovery (E) and validation (F) for the multivariate survival analysis in overall patients, missense mutation carriers, and other mutation carriers. **Figure S2.** Mutation frequency change of the DNA damage response pathway genes in presence of mutations in the 8-gene signature of mTOR pathway. The change of mutation frequency for each gene in DNA repair pathways in presence of the 8-gene signature mutation. Analysis was performed in TCGA (**A**) and was validated in MSKCC study (**B**), with frequency changes expressed as logarithmic ratio (mutant-type signature/wild-type signature patients). logarithmic *P* value indicated the level of significance, and those above the dashed line indicated statistical significance. **Figure S3.** Mutations and the activation status of mTOR pathway. Target gene of key mTOR pathway complement, including mTORC1 and mTORC2 (B). The association between the mutations in the 8-gene signature and mRNA expression of the target genes in response to mTOR pathway. **Figure S4.** Validation of the pathway enrichment analysis using transcriptome data from IMvigor210 clinical trial. Pathway enrichment analysis from IMvigor210 clinical trial using GSEA method, in the comparison of mutant-type versus wild-type patients for the 8-gene signature involved in mTOR pathway. We showed representative pathway enrichment involved in cancer immunity (A). All pathways enriched was summarized in one figure (B). We also summarized the enriched pathways overlapped with TCGA database using venn diagram and listed the overlapped in details (C). **Figure S5.** One clinical case with AKT1 mutation have good response to ICI treatment. An 82-year-old man with advanced squamous cell carcinoma of the lung was tested to have AKT1 mutation in tumors upon transbronchil biopsy. The tumor had shrunk by roughly two thirds after 2 cycle treatment of 200 mg pembrolizumab, as indicated by computed tomography images of plain film (A), mediastinal window (B) and pulmonary window (C). **Figure S6.** mTOR pathway mutations in circulating free DNA (cfDNA) and ICI treatment. Blood-based NGS was performed in OAK and POPLAR trial with comparison of ICI treatment versus chemotherapy, which was extracted for the mutation states of the 8-gene signature involved in mTOR pathway. The outcome comparison of atezolizumab versus docetaxel in patients with the 8-gene signature mutations in cfDNA, in terms of OS (A), PFS (B), and ORR (C) and DCB rate (D). **Figure S7.** The treatment outcome of patients with mTOR pathway mutations in circulating free DNA (cfDNA) in the subgroups stratified by TMB. The OS and PFS comparison of atezolizumab versus docetaxel in patients with the 8-gene signature mutations involved in mTOR pathway in cfDNA, with subgroup analysis in low-TMB patients (A-B) and high-TMB patients (C-D), respectively.

**Additional file 2: Table S1.** Patients and studies included for discovery and validation. **Table S2.** Demographics of patients in discovery stage. **Table S3.** Demographics of patients in validation stage. **Table S4.** mTOR pathway genes with mutation data available for analysis in TMB and immunotherapy study from MSKCC as discovery. **Table S5.** Frequency of DNA damage pathway genes mutation in the comparison of mTOR pathway wild-type versus mutant-type patients using sequencing data of 1661 cancer patients from MSKCC immunotherapy study. **Table S6.** Frequency of DNA damage pathway genes mutation in the comparison of mTOR pathway wild-type versus mutant-type patients using sequencing data of 10182 cancer patients from TCGA. **Table S7.** Pathway enriched in the comparison of mutant-type versus wild-type patients for the 8-gene signature involved in mTOR pathway in TCGA cancer tissues. **Table S8.** Pathway enriched in the comparison of mutant-type versus wild-type patients for the 8-gene signature involved in mTOR pathway in IMvigor210 study.

## Data Availability

The data and material were derived from cBioportal and TCGA dataset, which are publicly available at http://www.cbioportal.org and https://www.cancer.gov/about-nci/organization/ccg/research/structural-genomics/tcga.

## Abbreviations

ICI: Immune checkpoint inhibitors
mTORC1: MTOR complex1
cfDNA: Circulating free DNA
MSI: Microsatellite instability
HR: Hazard ratio
TMB: Tumor mutation burden
DDR: DNA damage response pathway
DSBR: DNA double-strand breaks repair
NER: Nucleotide excision repair
BER: Base excision repair
TCGA: The Cancer Genome Atlas
MSKCC: Memorial Sloan-Kettering Cancer Center
GSEA: Gene Set Enrichment Analysis
