# Supplementary material for: mTOR pathway gene mutations predict response to immune checkpoint inhibitors in multiple cancers
Source: J Transl Med. 2022 May 31;20:247. doi: 10.1186/s12967-022-03436-1 (PMC9153162; doi:10.1186/s12967-022-03436-1)
Supplement: Supplementary file 1 — Additional file 1: Figure S1. Mutation types of mTOR pathway and survival of patients with ICI treatment. The association of the missense mutations and other mutations in mTOR pathway with survival patients who underwent ICI treatment, respectively (A-B). The validation for this association stratified by mutation types (C-D). Visualization in discovery (E) and validation (F) for the multivariate survival analysis in overall patients, missense mutation carriers, and other mutation carriers. Figure S2. Mutation frequency change of the DNA damage response pathway genes in presence of mutations in the 8-gene signature of mTOR pathway. The change of mutation frequency for each gene in DNA repair pathways in presence of the 8-gene signature mutation. Analysis was performed in TCGA (A) and was validated in MSKCC study (B), with frequency changes expressed as logarithmic ratio (mutant-type signature/wild-type signature patients). logarithmic P value indicated the level of significance, and those above the dashed line indicated statistical significance. Figure S3. Mutations and the activation status of mTOR pathway. Target gene of key mTOR pathway complement, including mTORC1 and mTORC2 (B). The association between the mutations in the 8-gene signature and mRNA expression of the target genes in response to mTOR pathway. Figure S4. Validation of the pathway enrichment analysis using transcriptome data from IMvigor210 clinical trial. Pathway enrichment analysis from IMvigor210 clinical trial using GSEA method, in the comparison of mutant-type versus wild-type patients for the 8-gene signature involved in mTOR pathway. We showed representative pathway enrichment involved in cancer immunity (A). All pathways enriched was summarized in one figure (B). We also summarized the enriched pathways overlapped with TCGA database using venn diagram and listed the overlapped in details (C). Figure S5. One clinical case with AKT1 mutation have good response to ICI treatment. An 82-year-old [file 12967_2022_3436_MOESM1_ESM.pdf]

Figure S1

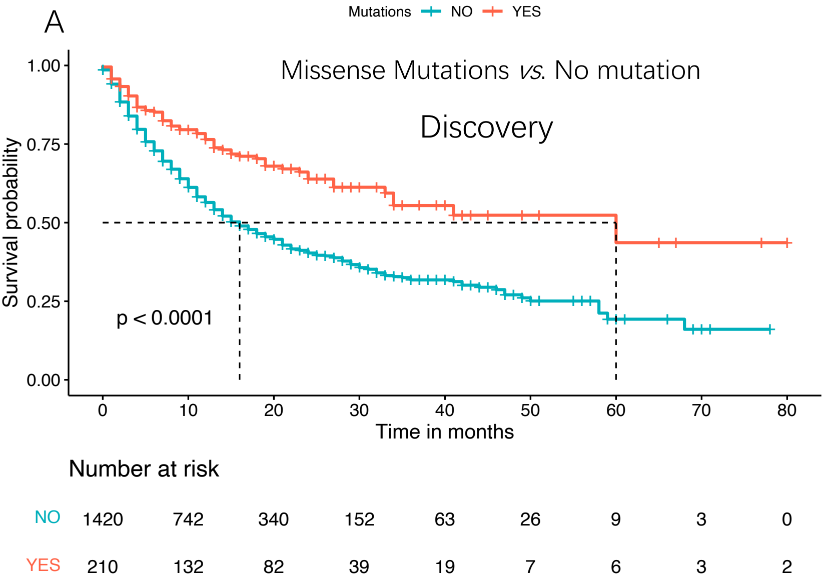

Comparison	HR (95% CI)	*P*
Yes *vs.* No	0.59 (0.45-0.77)	9.33×10^{-5}

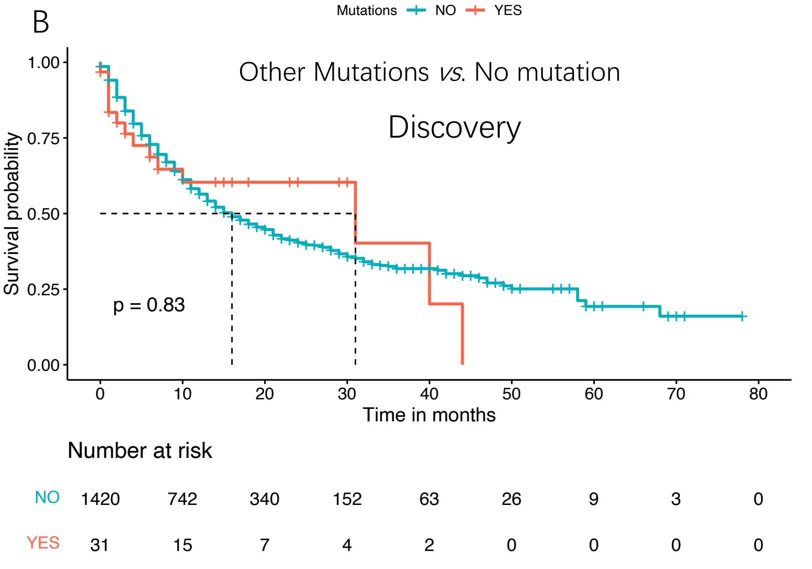

Comparison	HR (95% CI)	*P*
Yes *vs.* No	1.11 (0.65-1.91)	0.695

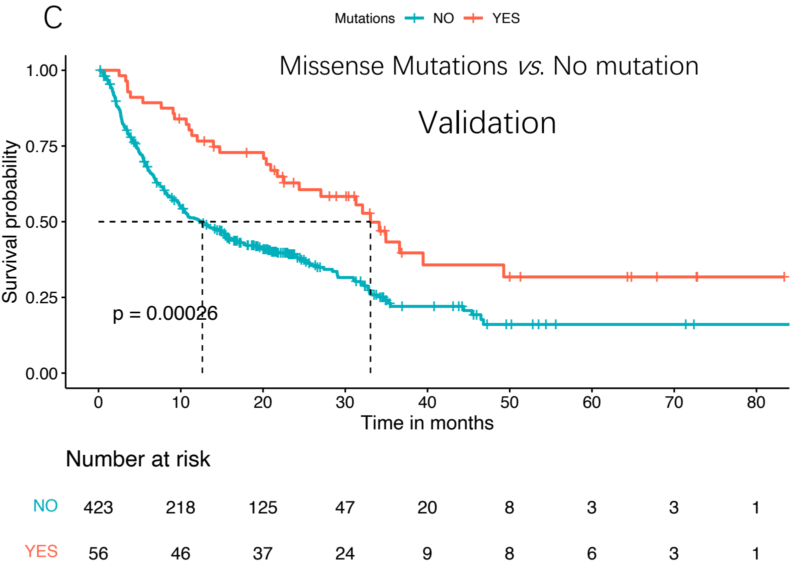

Comparison	LER (95% CI)	*P*
Yes *vs.* No	2.23 (1.43-3.49)	4.71×10^{-4}

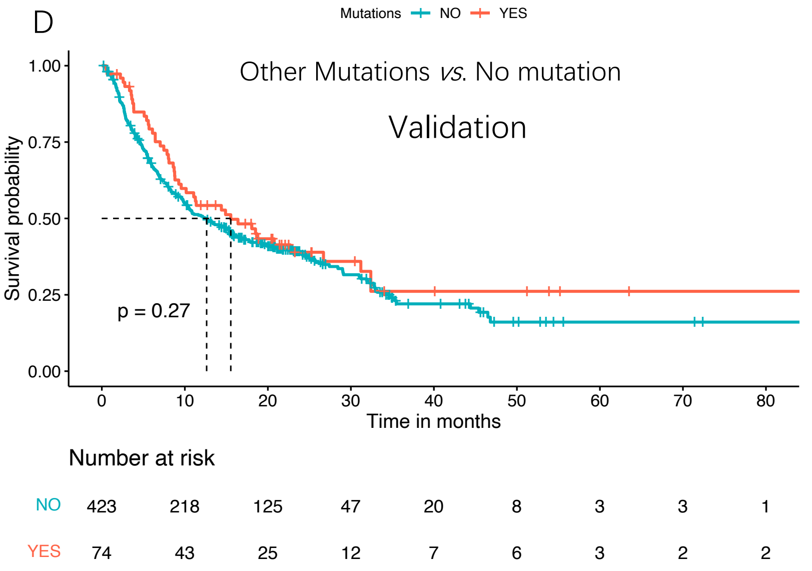

Comparison	LER (95% CI)	*P*
Yes *vs.* No	1.61 (0.96-2.72)	0.072

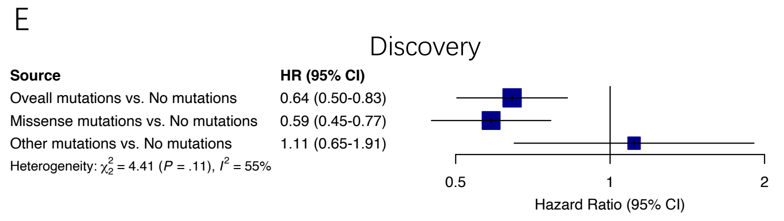

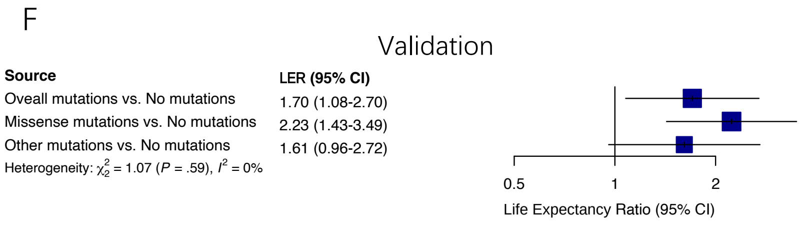

Figure S2

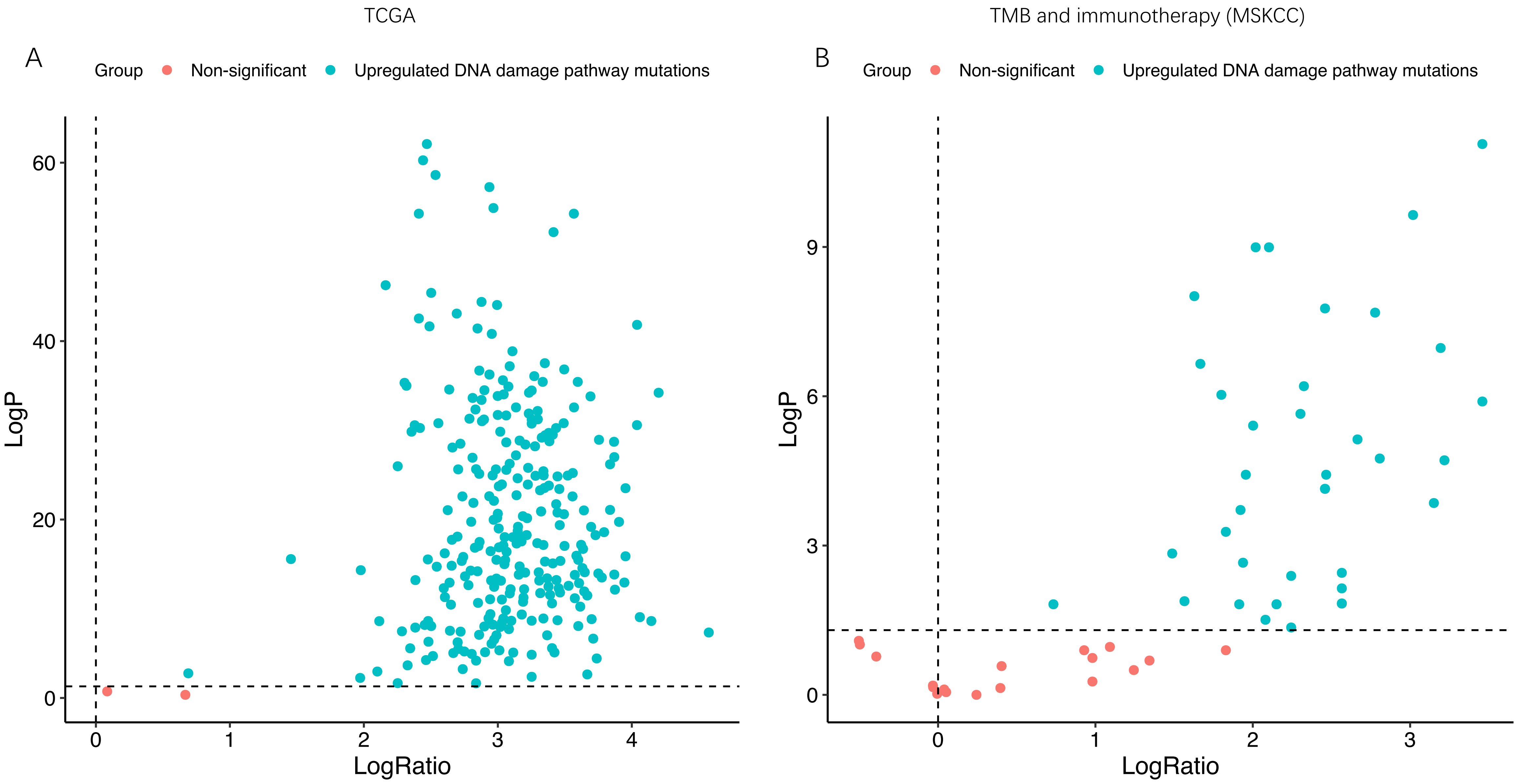
TCGA
A
Group
Non-significant
Upregulated DNA damage pathway mutations
LogP
LogRatio
TMB and immunotherapy (MSKCC)
B
Group
Non-significant
Upregulated DNA damage pathway mutations
LogP
LogRatio

Figure S3

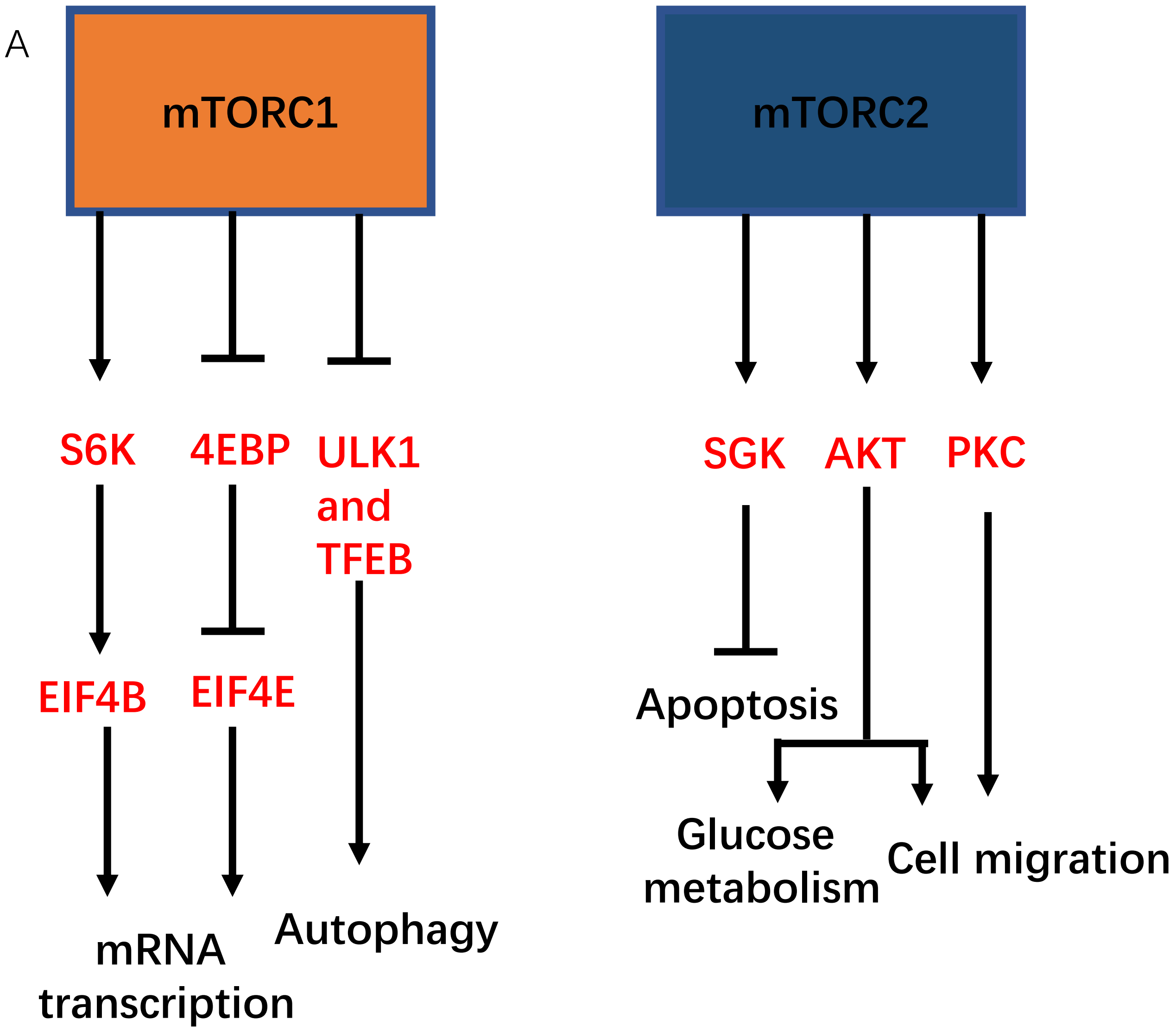
A
mTORC1
mTORC2
S6K
4EBP
ULK1 and TFEB
SGK
AKT
PKC
EIF4B
EIF4E
Apoptosis
Glucose metabolism
Cell migration
mRNA transcription
Autophagy

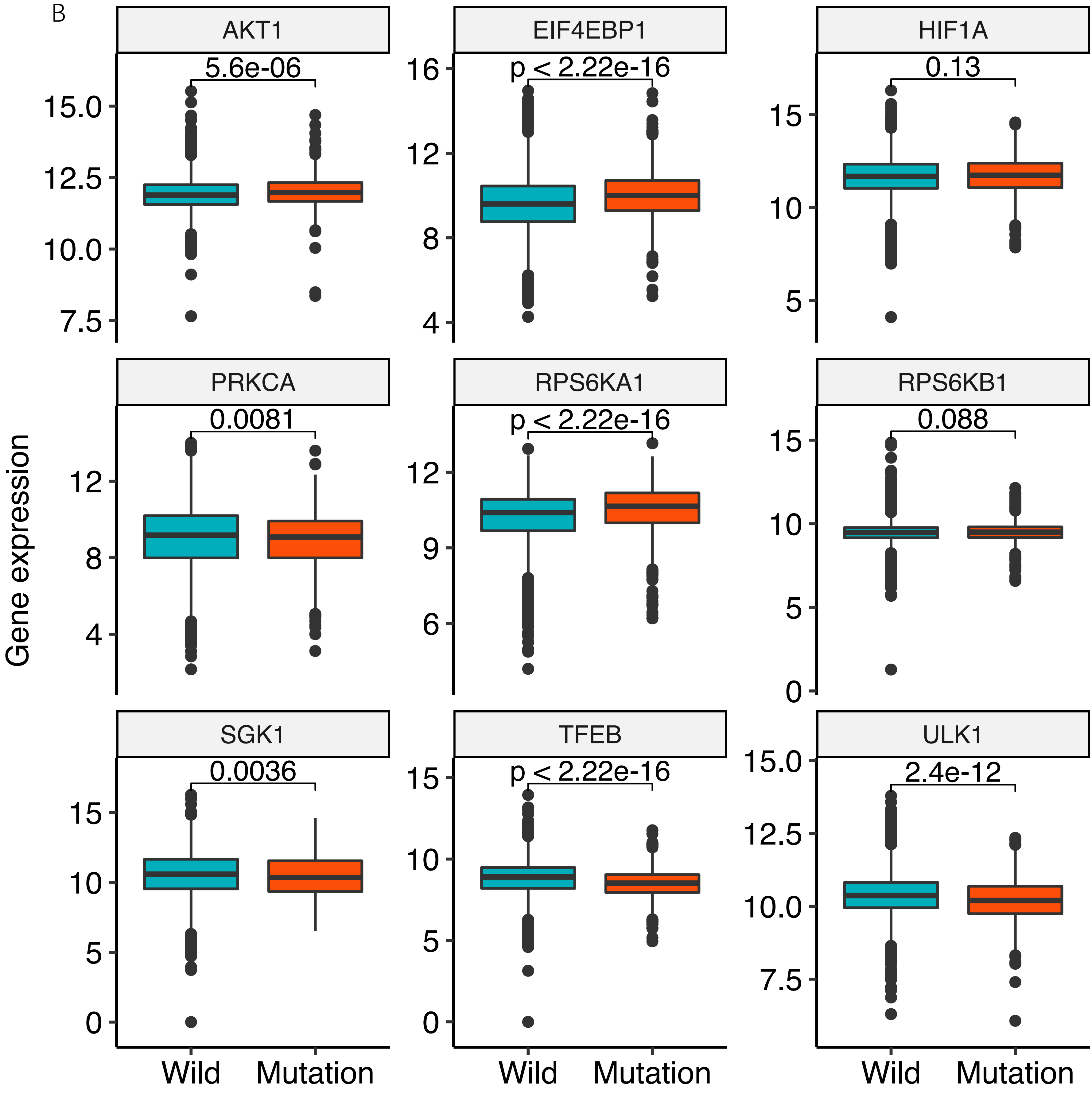
B
AKT1
5.6e-06
EIF4EBP1
p < 2.22e-16
HIF1A
0.13
PRKCA
0.0081
RPS6KA1
p < 2.22e-16
RPS6KB1
0.088
SGK1
0.0036
TFEB
p < 2.22e-16
ULK1
2.4e-12
Gene expression
Wild
Mutation

Figure S4

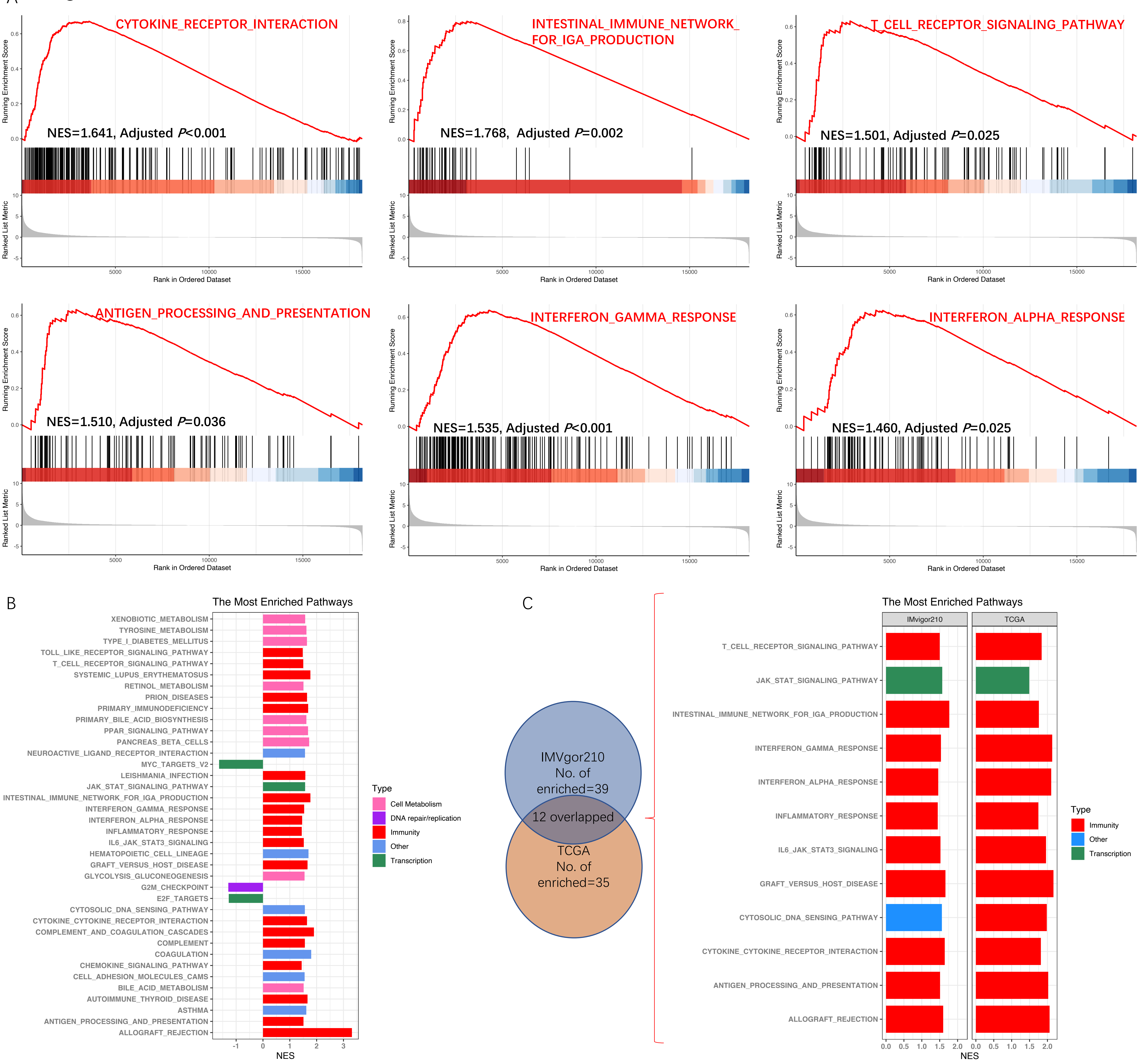
A
CYTOKINE_RECEPTOR_INTERACTION
NES=1.641, Adjusted P<0.001
INTESTINAL_IMMUNE_NETWORK_FOR_IGA_PRODUCTION
NES=1.768, Adjusted P=0.002
T_CELL_RECEPTOR_SIGNALING_PATHWAY
NES=1.501, Adjusted P=0.025
ANTIGEN_PROCESSING_AND_PRESENTATION
NES=1.510, Adjusted P=0.036
INTERFERON_GAMMA_RESPONSE
NES=1.535, Adjusted P<0.001
INTERFERON_ALPHA_RESPONSE
NES=1.460, Adjusted P=0.025
Running Enrichment Score
Ranked List Metric
Rank in Ordered Dataset
B
The Most Enriched Pathways
XENOBIOTIC_METABOLISM
TYROSINE_METABOLISM
TYPE_I_DIABETES_MELLITUS
TOLL_LIKE_RECEPTOR_SIGNALING_PATHWAY
T_CELL_RECEPTOR_SIGNALING_PATHWAY
SYSTEMIC_LUPUS_ERYTHEMATOSUS
RETINOL_METABOLISM
PRION_DISEASES
PRIMARY_IMMUNODEFICIENCY
PRIMARY_BILE_ACID_BIOSYNTHESIS
PPAR_SIGNALING_PATHWAY
PANCREAS_BETA_CELLS
NEUROACTIVE_LIGAND_RECEPTOR_INTERACTION
MYC_TARGETS_V2
LEISHMANIA_INFECTION
JAK_STAT_SIGNALING_PATHWAY
INTESTINAL_IMMUNE_NETWORK_FOR_IGA_PRODUCTION
INTERFERON_GAMMA_RESPONSE
INTERFERON_ALPHA_RESPONSE
INFLAMMATORY_RESPONSE
IL6_JAK_STAT3_SIGNALING
HEMATOPOIETIC_CELL_LINEAGE
GRAFT_VERSUS_HOST_DISEASE
GLYCOLYSIS_GLUCONEOGENESIS
G2M_CHECKPOINT
E2F_TARGETS
CYTOSOLIC_DNA_SENSING_PATHWAY
CYTOKINE_CYTOKINE_RECEPTOR_INTERACTION
COMPLEMENT_AND_COAGULATION_CASCADES
COMPLEMENT
COAGULATION
CHEMOKINE_SIGNALING_PATHWAY
CELL_ADHESION_MOLECULES_CAMS
BILE_ACID_METABOLISM
AUTOIMMUNE_THYROID_DISEASE
ASTHMA
ANTIGEN_PROCESSING_AND_PRESENTATION
ALLOGRAFT_REJECTION
NES
Type
Cell Metabolism
DNA repair/replication
Immunity
Other
Transcription
C
IMVgor210 No. of enriched=39
12 overlapped
TCGA No. of enriched=35
The Most Enriched Pathways
IMvigor210
TCGA
T_CELL_RECEPTOR_SIGNALING_PATHWAY
JAK_STAT_SIGNALING_PATHWAY
INTESTINAL_IMMUNE_NETWORK_FOR_IGA_PRODUCTION
INTERFERON_GAMMA_RESPONSE
INTERFERON_ALPHA_RESPONSE
INFLAMMATORY_RESPONSE
IL6_JAK_STAT3_SIGNALING
GRAFT_VERSUS_HOST_DISEASE
CYTOSOLIC_DNA_SENSING_PATHWAY
CYTOKINE_CYTOKINE_RECEPTOR_INTERACTION
ANTIGEN_PROCESSING_AND_PRESENTATION
ALLOGRAFT_REJECTION
NES
Type
Immunity
Other
Transcription

Figure S5

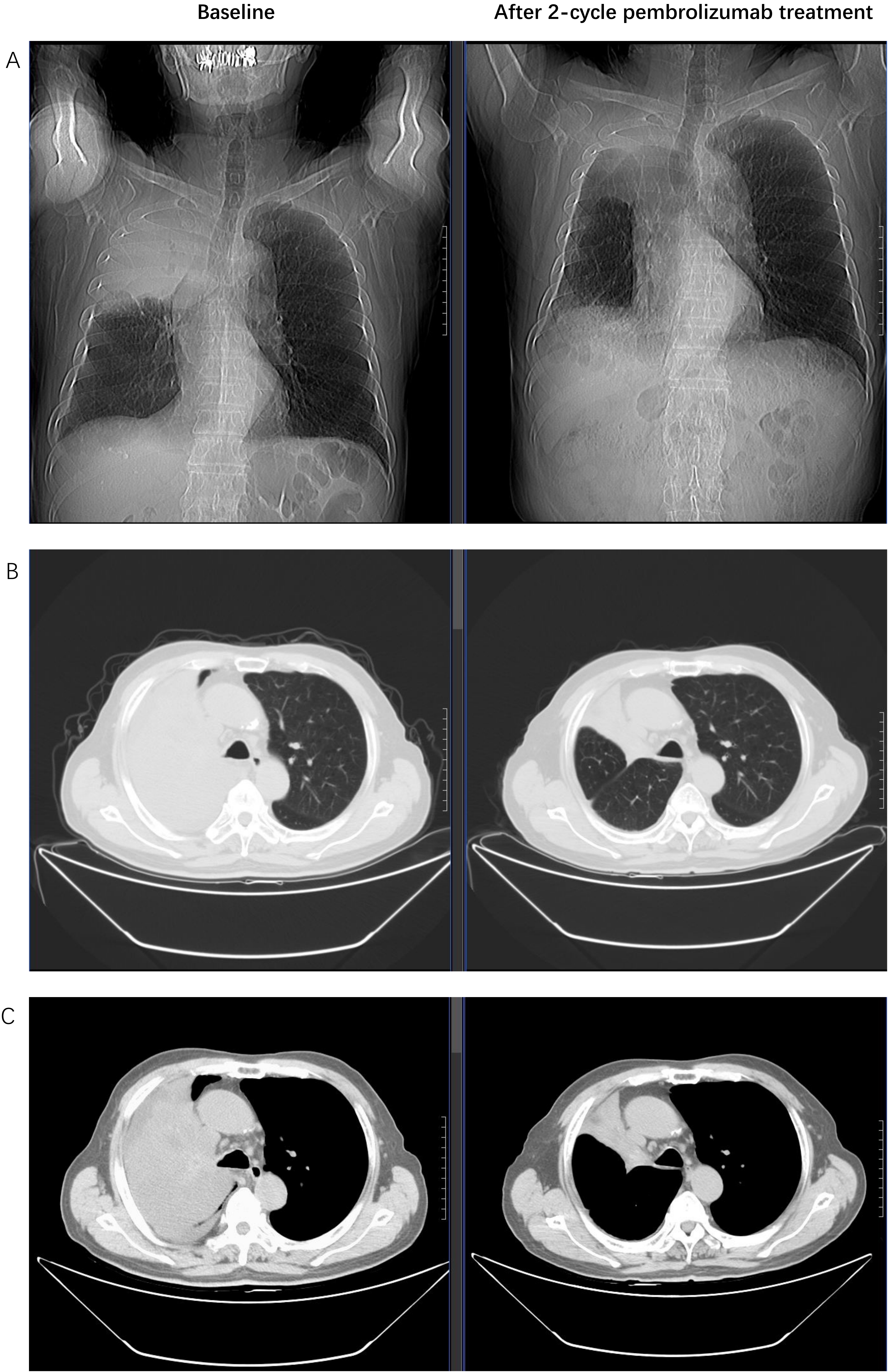

Figure S6

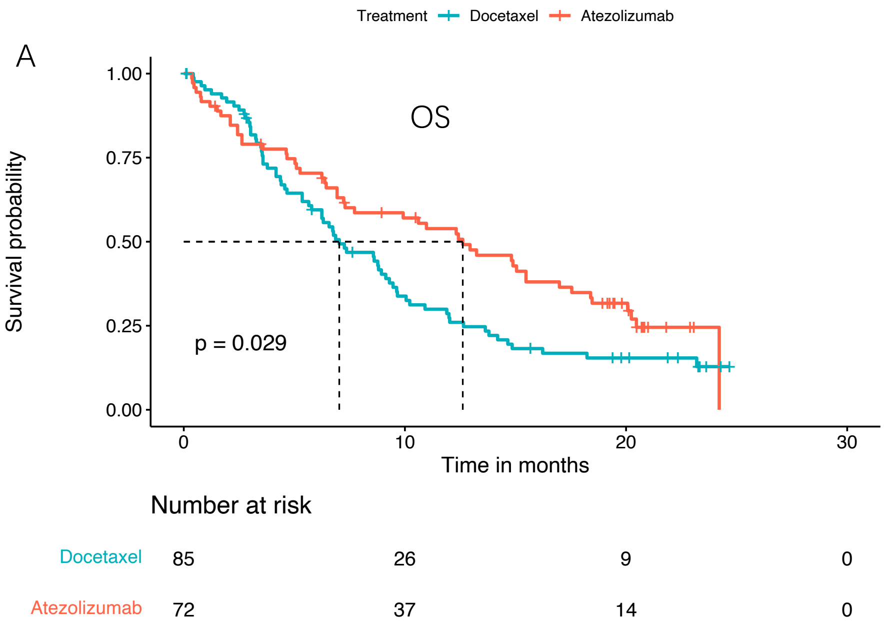

Comparison	HR (95% CI)	*P*
Atezo *vs.* Dox	0.57 (0.38-0.84)	0.005

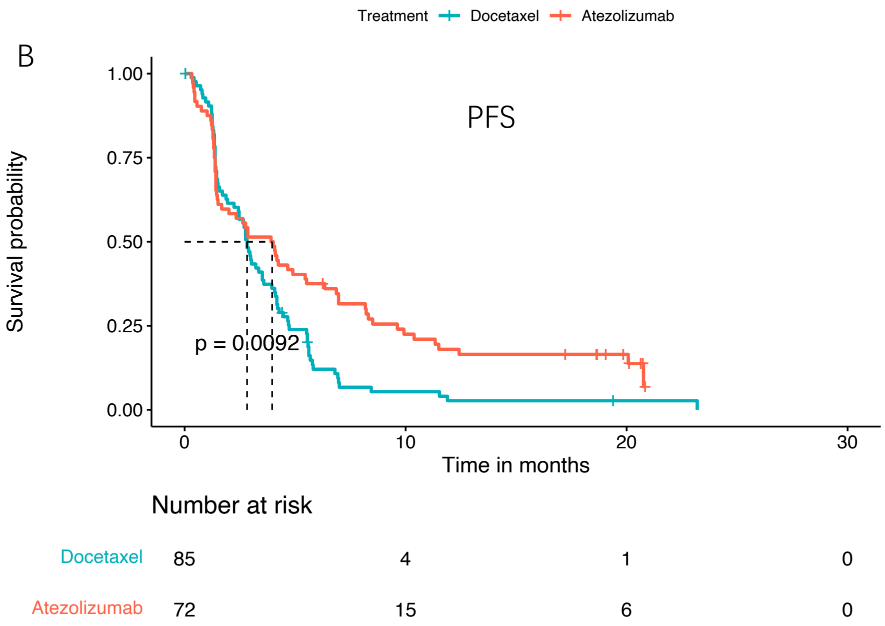

Comparison	HR (95% CI)	*P*
Atezo *vs.* Dox	0.60 (0.42-0.87)	0.007

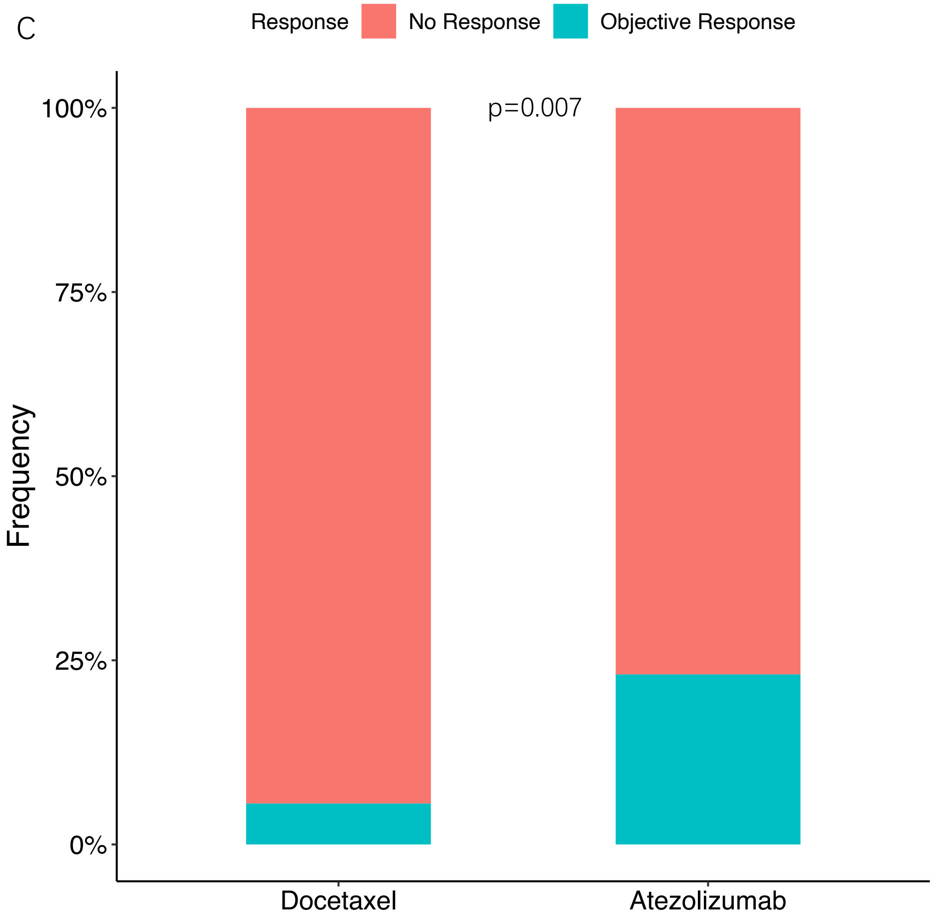

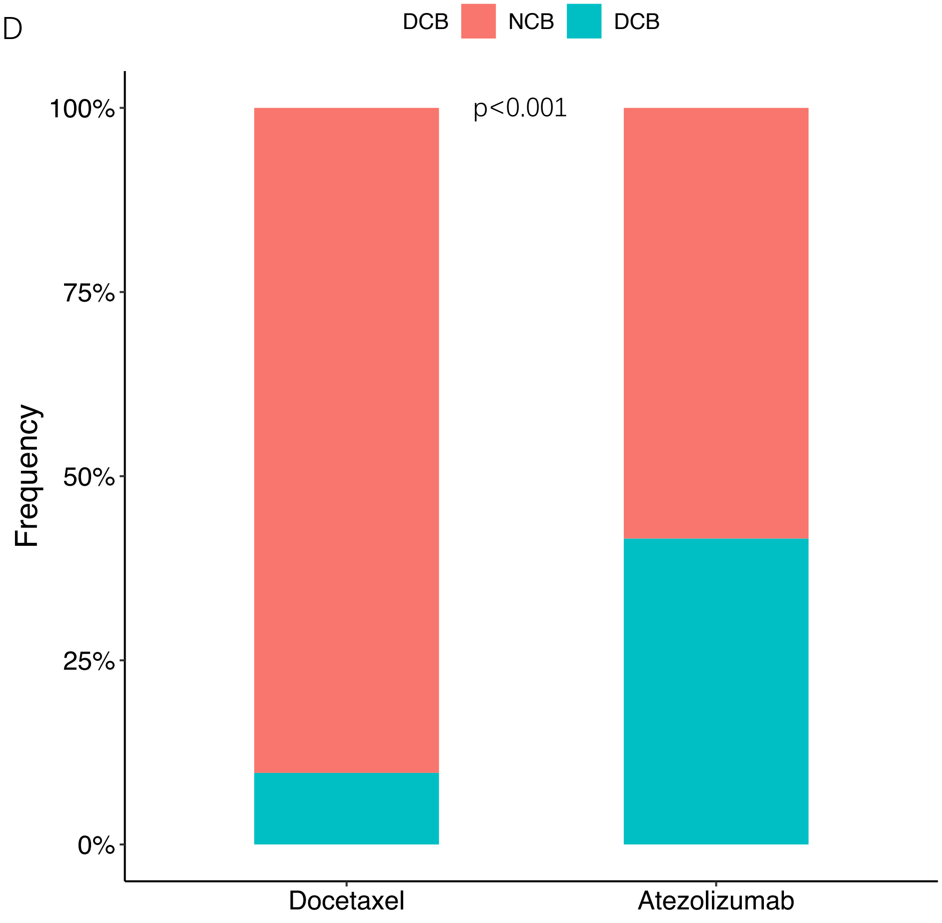

Figure S7

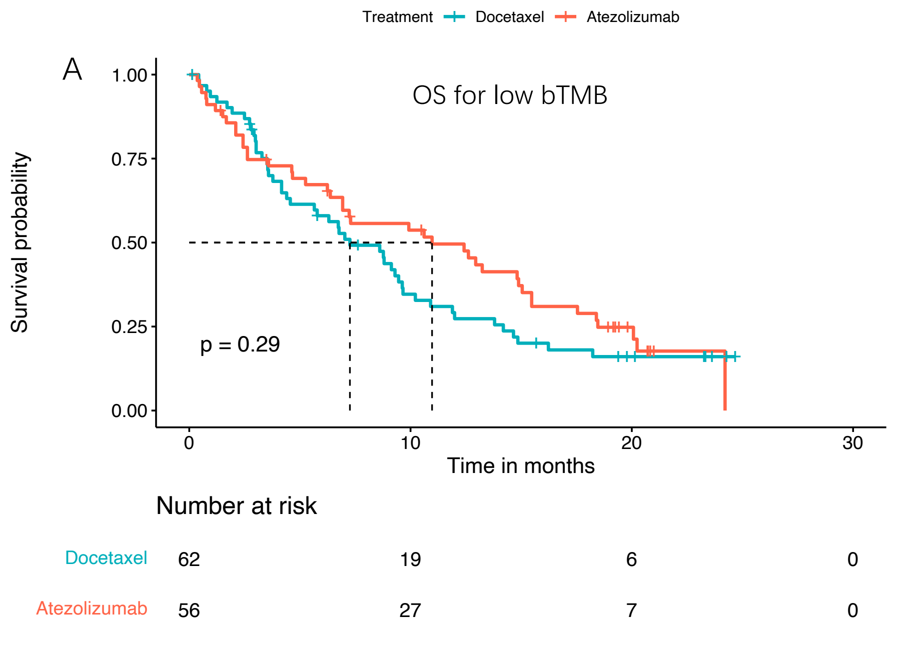

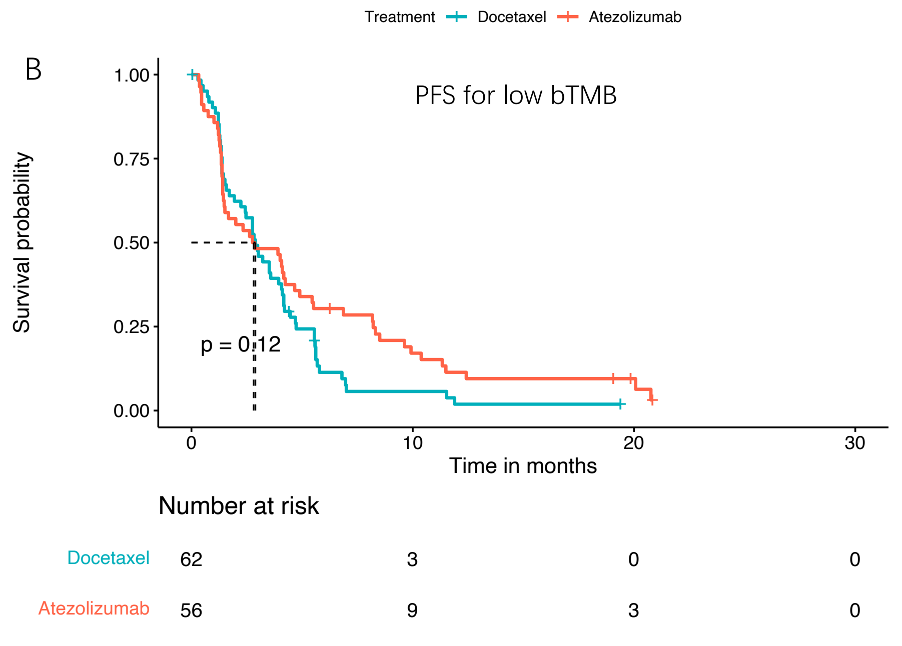

**Comparison**	**HR (95% CI)**	***P***
Atezo *vs*. Dox	0.60 (0.38-0.96)	0.033

**Comparison**	**LER (95% CI)**	***P***
Atezo *vs*. Dox	1.51 (1,02-2.24)	0.040

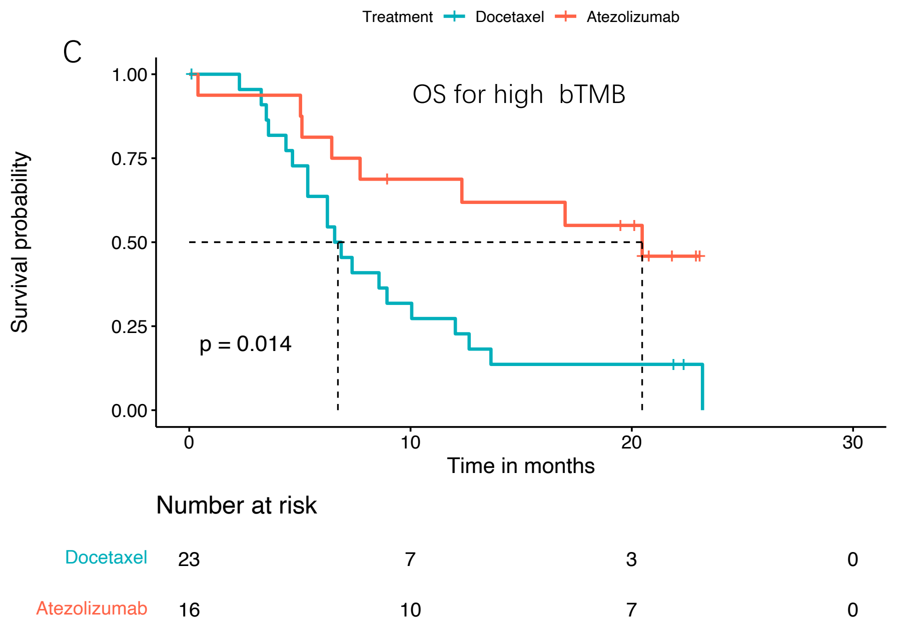

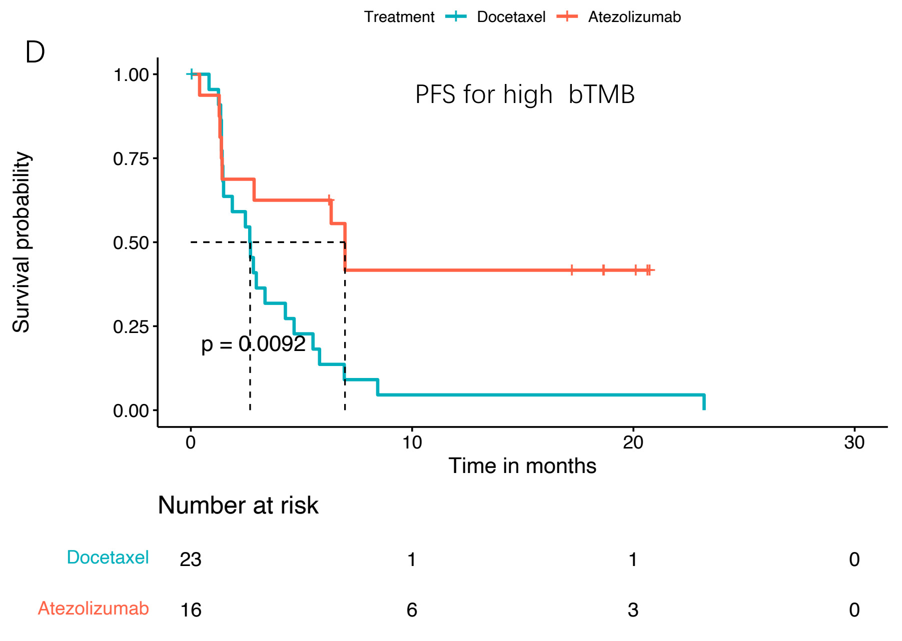

**Comparison**	**HR (95% CI)**	***P***
Atezo *vs*. Dox	0.36 (0.15-0.88)	0.026

**Comparison**	**LER (95% CI)**	***P***
Atezo *vs*. Dox	1.46 (0.75-2.88)	0.263
