# Supplementary material for: mTOR pathway gene mutations predict response to immune checkpoint inhibitors in multiple cancers
Source: J Transl Med. 2022 May 31;20:247. doi: 10.1186/s12967-022-03436-1 (PMC9153162; doi:10.1186/s12967-022-03436-1)
Supplement: Supplementary file 2 — Additional file 2: Table S1. Patients and studies included for discovery and validation. Table S2. Demographics of patients in discovery stage. Table S3. Demographics of patients in validation stage. Table S4. mTOR pathway genes with mutation data available for analysis in TMB and immunotherapy study from MSKCC as discovery. Table S5. Frequency of DNA damage pathway genes mutation in the comparison of mTOR pathway wild-type versus mutant-type patients using sequencing data of 1661 cancer patients from MSKCC immunotherapy study. Table S6. Frequency of DNA damage pathway genes mutation in the comparison of mTOR pathway wild-type versus mutant-type patients using sequencing data of 10182 cancer patients from TCGA. Table S7. Pathway enriched in the comparison of mutant-type versus wild-type patients for the 8-gene signature involved in mTOR pathway in TCGA cancer tissues. Table S8. Pathway enriched in the comparison of mutant-type versus wild-type patients for the 8-gene signature involved in mTOR pathway in IMvigor210 study. [file 12967_2022_3436_MOESM2_ESM.docx]

| **Table S1:** Patients and studies included for discovery and validation | | | | | |
| --- | --- | --- | --- | --- | --- |
| Stage | Study | Year | No. of Patients | Source | Total Patients |
| Discvoery | TMB and immunotherapy (MSKCC) | 2019 | 1661 | NGS | 1661 |
| Validation | CCRCC (DFCI) | 2019 | 35 | WES | 553 |
|  | GBM (Columbia) | 2019 | 32 | WES |  |
|  | MEL (ULCA) | 2016 | 37 | WES |  |
|  | SKCM (DFCI) | 2015 | 110 | WES |  |
|  | SKCM (MSKCC) | 2014 | 64 | WES |  |
|  | IMvigor210 (Italy) | 2018 | 275 | NGS |  |
| Abbreviations: TMB, Tumor mutation burden; WES, Whole exome sequencing; NGS, Next-generation sequencing; CCRCC, Clear cell renal cell carcinoma; GBM, Glioblastoma multiforme; MEL, Melanoma; SKCM, Skin cutaneous melanoma; MSKCC, Memorial sloan-kettering cancer center; DFCI, Dana-farber cancer institute; ULCA, University of California. | | | | | |

`

| **Table S2:** Demographics of patients in discovery stage | | |
| --- | --- | --- |
| Variable | No. of patients (%) | No. of deaths  (%) |
|  |  |  |
| **Age** |  |  |
| ≤60 | 1162 | 585 |
| >60 | 499 | 247 |
| **Gender** |  |  |
| Female | 627 | 324 |
| Male | 1034 | 508 |
| **Treatment** |  |  |
| CTLA4 | 99 | 54 |
| PD-1/PD-L1 | 1307 | 682 |
| Como | 255 | 96 |
| **Cancer type** |  |  |
| BLCA | 215 | 95 |
| BRCA | 44 | 31 |
| UPCA | 88 | 36 |
| CRC | 110 | 45 |
| ESCC | 126 | 57 |
| Glioma | 117 | 87 |
| HNSC | 139 | 79 |
| Melanoma | 320 | 125 |
| NSCLC | 350 | 219 |
| RCC | 151 | 58 |
| SKC | 1 | 0 |
| **TMB** |  |  |
| ≤10/Mb | 1173 | 651 |
| >10/Mb | 488 | 181 |
| Abbreviations: Ipi, Ipilimumab; Nivo, Nivolumab; Pemb, Pembrolizumab; Treme; Tremelimumab; Atezo, Atezolizumab; BLCA, Bladder Urothelial Carcinoma; BRCA, Breast invasive carcinoma; UPCA, Cancer of Unknown Primary; CRC, Colorectal cancer; ESCC, Esophagogastric Cancer; HNSC, Head and Neck Cancer; NSCLC, Non-Small Cell Lung Cancer; RCC, Renal cell carcinoma; SKC, Skin cancer; SKC, Skin Cancer, Non-Melanoma; TMB, Tumor mutation burden | | |

| **Table S3:** Demographics of patients in validation stage | | |
| --- | --- | --- |
| Variable | No. of patients (%) | No. of deaths  (%) |
|  |  |  |
| **Age** |  |  |
| ≤60 | 131 | 76 |
| >60 | 147 | 93 |
| NA | 275 | 177 |
| **Gender** |  |  |
| Female | 148 | 99 |
| Male | 405 | 247 |
| **Treatment** |  |  |
| Ipi | 170 | 113 |
| Nivo | 63 | 30 |
| Pemb | 41 | 23 |
| Treme | 4 | 3 |
| Atezo | 275 | 177 |
| **Cancer type** |  |  |
| Glioma | 32 | 11 |
| Melanoma | 211 | 135 |
| RCC | 35 | 23 |
| UC | 275 | 177 |
| **TMB** |  |  |
| Low TMB | 327 | 228 |
| High TMB | 155 | 80 |
| Abbreviations: Ipi, Ipilimumab; Nivo, Nivolumab; Pemb, Pembrolizumab; Treme; Tremelimumab; Atezo, Atezolizumab; RCC, Renal cell carcinoma; UC, Urothelial carcinoma; TMB, Tumor mutation burden  **^a^** with adjustment for age, gender, treatment, cancer type and TMB | | |

| **Table S4:** mTOR pathway genes with mutation data available for analysis in TMB and immunotherapy study from MSKCC as discovery | | |
| --- | --- | --- |
| Gene name | Call rate | Mutation frequency |
| FGFR2 | 100% | 3.07% |
| IRS1 | 100% | 2.95% |
| PIK3R1 | 100% | 2.83% |
| IRS2 | 100% | 2.83% |
| PIK3C3 | 100% | 2.41% |
| FGFR4 | 100% | 1.87% |
| FGFR1 | 100% | 1.38% |
| FGF19 | 100% | 0.72% |
| FGF3 | 100% | 1.20% |
| RICTOR | 100% | 2.77% |
| RAC1 | 100% | 1.93% |
| AKT1 | 100% | 1.38% |
| MTOR | 100% | 4.39% |
| TSC1 | 100% | 3.79% |
| RPTOR | 100% | 2.89% |
| PIK3CA | 100% | 12.04% |
| FGFR3 | 100% | 4.09% |
| PIK3CB | 100% | 2.47% |
| PIK3R2 | 100% | 1.32% |
| PTPN11 | 100% | 1.26% |
| FGF4 | 100% | 1.02% |
| TSC2 | 100% | 3.85% |
| AKT2 | 100% | 1.14% |

| **Table S5:** Frequency of DNA damage pathway genes mutation in the comparison of mTOR pathway wild-type versus mutant-type patients using sequencing data of 1661 cancer patients from MSKCC immunotherapy study | | | | | | | | |
| --- | --- | --- | --- | --- | --- | --- | --- | --- |
| NAME | Freq (W) | Freq (M) | Ratio (M/W) | *P* | *P* Adj | Log(P) | Log(Ratio) | Group |
| ATM | 0.049 | 0.154 | 3.175 | 3.52E-08 | 2.23E-07 | 6.651 | 1.667 | Upregulated DNA damage pathway mutations |
| ATR | 0.036 | 0.125 | 3.483 | 1.80E-07 | 9.35E-07 | 6.029 | 1.800 | Upregulated DNA damage pathway mutations |
| AURKA | 0.008 | 0.029 | 3.768 | 0.009 | 0.015 | 1.820 | 1.914 | Non-significant |
| AURKB | 0.004 | 0.017 | 4.737 | 0.030 | 0.044 | 1.353 | 2.244 | Non-significant |
| BLM | 0.013 | 0.092 | 6.856 | 2.56E-09 | 2.09E-08 | 7.680 | 2.777 | Upregulated DNA damage pathway mutations |
| BRCA1 | 0.024 | 0.096 | 4.005 | 9.55E-07 | 3.89E-06 | 5.410 | 2.002 | Upregulated DNA damage pathway mutations |
| BRCA2 | 0.040 | 0.163 | 4.051 | 7.13E-11 | 1.02E-09 | 8.993 | 2.018 | Upregulated DNA damage pathway mutations |
| BRIP1 | 0.018 | 0.067 | 3.789 | 7.79E-05 | 1.930E-04 | 3.715 | 1.922 | Upregulated DNA damage pathway mutations |
| CCND1 | 0.004 | 0.008 | 2.368 | 0.268 | 0.318 | 0.497 | 1.244 | Non-significant |
| CCND2 | 0.005 | 0.021 | 4.229 | 0.020 | 0.031 | 1.508 | 2.080 | Non-significant |
| CCND3 | 0.002 | 0.004 | 1.974 | 0.465 | 0.541 | 0.267 | 0.981 | Non-significant |
| CCNE1 | 0.004 | 0.025 | 5.921 | 0.004 | 0.007 | 2.140 | 2.566 | Non-significant |
| CDC73 | 0.005 | 0.054 | 10.996 | 2.68E-07 | 1.27E-06 | 5.895 | 3.459 | Upregulated DNA damage pathway mutations |
| CDK12 | 0.020 | 0.079 | 3.879 | 1.26E-05 | 3.77E-05 | 4.423 | 1.956 | Upregulated DNA damage pathway mutations |
| CDK4 | 0.006 | 0.025 | 4.441 | 0.009 | 0.015 | 1.820 | 2.151 | Non-significant |
| CDK6 | 0.004 | 0.021 | 5.921 | 0.008 | 0.015 | 1.836 | 2.566 | Non-significant |
| CDK8 | 0.004 | 0.021 | 5.921 | 0.008 | 0.015 | 1.836 | 2.566 | Non-significant |
| CDKN2C | 0.006 | 0.008 | 1.316 | 0.666 | 0.730 | 0.137 | 0.396 | Non-significant |
| CENPA | 0.165 | 0.117 | 0.705 | 0.056 | 0.082 | 1.085 | -0.503 | Non-significant |
| CHEK1 | 0.004 | 0.038 | 8.881 | 5.39E-05 | 0.000139751 | 3.855 | 3.151 | Upregulated DNA damage pathway mutations |
| CHEK2 | 0.012 | 0.046 | 3.831 | 0.001 | 0.002 | 2.655 | 1.938 | Non-significant |
| ERCC2 | 0.018 | 0.038 | 2.132 | 0.078 | 0.109 | 0.963 | 1.092 | Non-significant |
| ERCC4 | 0.014 | 0.042 | 2.960 | 0.007 | 0.013 | 1.882 | 1.566 | Non-significant |
| FANCA | 0.018 | 0.063 | 3.553 | 2.23E-04 | 0.001 | 3.276 | 1.829 | Upregulated DNA damage pathway mutations |
| H3C1 | 0.165 | 0.117 | 0.708 | 0.068 | 0.097 | 1.011 | -0.497 | Non-significant |
| LATS1 | 0.018 | 0.092 | 5.010 | 1.10E-07 | 6.27E-07 | 6.203 | 2.325 | Upregulated DNA damage pathway mutations |
| LATS2 | 0.017 | 0.083 | 4.934 | 5.15E-07 | 2.26E-06 | 5.647 | 2.303 | Upregulated DNA damage pathway mutations |
| MDM2 | 0.006 | 0.025 | 4.441 | 0.009 | 0.015 | 1.820 | 2.151 | Non-significant |
| MLH1 | 0.008 | 0.071 | 9.150 | 1.51E-08 | 1.07E-07 | 6.970 | 3.194 | Upregulated DNA damage pathway mutations |
| MRE11 | 0.010 | 0.054 | 5.498 | 2.67E-05 | 7.25E-05 | 4.140 | 2.459 | Upregulated DNA damage pathway mutations |
| MSH2 | 0.010 | 0.108 | 10.996 | 1.50E-13 | 8.54E-12 | 11.069 | 3.459 | Upregulated DNA damage pathway mutations |
| MSH6 | 0.013 | 0.108 | 8.102 | 7.99E-12 | 2.28E-10 | 9.643 | 3.018 | Upregulated DNA damage pathway mutations |
| MUTYH | 0.007 | 0.033 | 4.737 | 0.002 | 0.004 | 2.390 | 2.244 | Non-significant |
| BABAM1 | 0.754 | 0.738 | 0.979 | 0.628 | 0.702 | 0.154 | -0.031 | Non-significant |
| NBN | 0.011 | 0.058 | 5.526 | 1.23E-05 | 3.77E-05 | 4.423 | 2.466 | Upregulated DNA damage pathway mutations |
| NPM1 | 0.004 | 0.004 | 1.184 | 1 | 1 | 0.000 | 0.244 | Non-significant |
| CDKN2A | 0.080 | 0.133 | 1.662 | 0.010 | 0.015 | 1.819 | 0.733 | Non-significant |
| CDKN1A | 0.020 | 0.038 | 1.903 | 0.096 | 0.127 | 0.896 | 0.928 | Non-significant |
| CDKN1B | 0.004 | 0.013 | 3.553 | 0.096 | 0.127 | 0.896 | 1.829 | Non-significant |
| TP53 | 0.446 | 0.458 | 1.027 | 0.726 | 0.781 | 0.107 | 0.039 | Non-significant |
| TP53..MUT | 0.443 | 0.454 | 1.026 | 0.779 | 0.822 | 0.085 | 0.037 | Non-significant |
| TP63 | 0.036 | 0.154 | 4.296 | 6.99E-11 | 1.02E-09 | 8.993 | 2.103 | Upregulated DNA damage pathway mutations |
| PLK2 | 0.013 | 0.025 | 1.974 | 0.143 | 0.182 | 0.741 | 0.981 | Non-significant |
| PPM1D | 0.169 | 0.175 | 1.036 | 0.853 | 0.884 | 0.054 | 0.051 | Non-significant |
| PPP2R1A | 0.008 | 0.054 | 6.997 | 5.00E-06 | 1.78E-05 | 4.749 | 2.807 | Upregulated DNA damage pathway mutations |
| RAD21 | 0.170 | 0.129 | 0.762 | 0.131 | 0.170 | 0.769 | -0.393 | Non-significant |
| RAD50 | 0.010 | 0.054 | 5.498 | 2.67E-05 | 7.25E-05 | 4.140 | 2.459 | Upregulated DNA damage pathway mutations |
| RAD51 | 0.005 | 0.013 | 2.538 | 0.165 | 0.205 | 0.689 | 1.343 | Non-significant |
| RAD54L | 0.005 | 0.029 | 5.921 | 0.002 | 0.004 | 2.451 | 2.566 | Non-significant |
| RB1 | 0.066 | 0.088 | 1.323 | 0.218 | 0.265 | 0.577 | 0.404 | Non-significant |
| RECQL | 0.753 | 0.750 | 0.996 | 0.936 | 0.952 | 0.021 | -0.006 | Non-significant |
| RECQL4 | 0.020 | 0.108 | 5.498 | 1.81E-09 | 1.72E-08 | 7.765 | 2.459 | Upregulated DNA damage pathway mutations |
| SESN2 | 0.754 | 0.738 | 0.978 | 0.572 | 0.652 | 0.185 | -0.033 | Non-significant |
| SMARCA4 | 0.063 | 0.196 | 3.092 | 8.54E-10 | 9.73E-09 | 8.012 | 1.629 | Upregulated DNA damage pathway mutations |
| STAG2 | 0.027 | 0.075 | 2.805 | 0.001 | 0.001 | 2.840 | 1.488 | Upregulated DNA damage pathway mutations |
| TOP1 | 0.010 | 0.063 | 6.344 | 1.94E-06 | 7.36E-06 | 5.133 | 2.665 | Upregulated DNA damage pathway mutations |
| ERCC3 | 0.005 | 0.046 | 9.304 | 5.77E-06 | 1.94E-05 | 4.713 | 3.218 | Upregulated DNA damage pathway mutations |
| Abbreviation: Freq, Frequency of DNA damage pathway genes mutation; W, mTOR pathway wild-type patients; M, mTOR pathway mutant-type patients; *P*, *P* value; *P* Adj, Bonferroni-adjusted *P* value. | | | | | | | | |

| **Table S6**: Frequency of DNA damage pathway genes mutation in the comparison of mTOR pathway wild-type versus mutant-type patients using sequencing data of 10182 cancer patients from TCGA | | | | | | | | |
| --- | --- | --- | --- | --- | --- | --- | --- | --- |
| NAME | Freq (W) | Freq (M) | Ratio (M/W) | P | P Adj | Log(P) | Log(Ratio) | Group |
| ACD | 0.004 | 0.035 | 8.308 | 1.96E-16 | 3.45E-16 | 15.462 | 3.054 | Upregulated DNA damage pathway mutations |
| ADD1 | 0.006 | 0.048 | 7.828 | 4.61E-21 | 1.07E-20 | 19.972 | 2.969 | Upregulated DNA damage pathway mutations |
| ALKBH7 | 0.001 | 0.009 | 9.529 | 1.34E-05 | 1.41E-05 | 4.849 | 3.252 | Upregulated DNA damage pathway mutations |
| ANAPC1 | 0.012 | 0.089 | 7.659 | 4.88E-38 | 5.56E-37 | 36.255 | 2.937 | Upregulated DNA damage pathway mutations |
| ANAPC10 | 0.001 | 0.013 | 17.698 | 1.95E-09 | 2.36E-09 | 8.627 | 4.145 | Upregulated DNA damage pathway mutations |
| ANAPC11 | 0.000 | 0.004 | 12.706 | 0.002 | 0.002 | 2.641 | 3.667 | Upregulated DNA damage pathway mutations |
| ANAPC2 | 0.006 | 0.040 | 6.299 | 8.85E-16 | 1.50E-15 | 14.823 | 2.655 | Upregulated DNA damage pathway mutations |
| ANP32A | 0.002 | 0.019 | 8.577 | 1.81E-09 | 2.21E-09 | 8.655 | 3.100 | Upregulated DNA damage pathway mutations |
| APEX1 | 0.002 | 0.021 | 10.588 | 1.88E-11 | 2.47E-11 | 10.607 | 3.404 | Upregulated DNA damage pathway mutations |
| APTX | 0.002 | 0.023 | 10.482 | 2.06E-12 | 2.83E-12 | 11.549 | 3.390 | Upregulated DNA damage pathway mutations |
| ARPP19 | 0.000 | 0.010 | 23.824 | 4.01E-08 | 4.59E-08 | 7.338 | 4.574 | Upregulated DNA damage pathway mutations |
| ATM | 0.038 | 0.169 | 4.476 | 1.71E-48 | 5.41E-47 | 46.267 | 2.162 | Upregulated DNA damage pathway mutations |
| ATR | 0.024 | 0.119 | 4.936 | 5.00E-37 | 4.75E-36 | 35.323 | 2.303 | Upregulated DNA damage pathway mutations |
| ATRAID | 0.001 | 0.016 | 12.995 | 1.18E-09 | 1.46E-09 | 8.836 | 3.700 | Upregulated DNA damage pathway mutations |
| ATRIP | 0.005 | 0.049 | 9.953 | 1.92E-24 | 5.25E-24 | 23.280 | 3.315 | Upregulated DNA damage pathway mutations |
| AURKA | 0.002 | 0.024 | 9.963 | 1.27E-12 | 1.76E-12 | 11.754 | 3.317 | Upregulated DNA damage pathway mutations |
| AURKB | 0.002 | 0.026 | 10.829 | 3.95E-14 | 6.05E-14 | 13.219 | 3.437 | Upregulated DNA damage pathway mutations |
| AURKC | 0.005 | 0.039 | 8.230 | 4.03E-18 | 7.92E-18 | 17.101 | 3.041 | Upregulated DNA damage pathway mutations |
| BABAM1 | 0.002 | 0.018 | 9.529 | 1.78E-09 | 2.19E-09 | 8.660 | 3.252 | Upregulated DNA damage pathway mutations |
| BLM | 0.011 | 0.078 | 7.368 | 1.80E-32 | 9.52E-32 | 31.022 | 2.881 | Upregulated DNA damage pathway mutations |
| BORA | 0.004 | 0.032 | 8.952 | 1.05E-15 | 1.78E-15 | 14.750 | 3.162 | Upregulated DNA damage pathway mutations |
| BRCA1 | 0.019 | 0.090 | 4.765 | 3.05E-27 | 1.06E-26 | 25.975 | 2.252 | Upregulated DNA damage pathway mutations |
| BRCA2 | 0.030 | 0.172 | 5.794 | 2.52E-61 | 2.40E-59 | 58.621 | 2.535 | Upregulated DNA damage pathway mutations |
| BRCC3 | 0.003 | 0.031 | 10.210 | 2.98E-16 | 5.15E-16 | 15.288 | 3.352 | Upregulated DNA damage pathway mutations |
| BRD2 | 0.008 | 0.047 | 5.569 | 1.64E-16 | 2.94E-16 | 15.531 | 2.477 | Upregulated DNA damage pathway mutations |
| BRIP1 | 0.012 | 0.078 | 6.325 | 2.26E-29 | 8.46E-29 | 28.073 | 2.661 | Upregulated DNA damage pathway mutations |
| BRSK1 | 0.008 | 0.060 | 7.785 | 3.57E-26 | 1.12E-25 | 24.951 | 2.961 | Upregulated DNA damage pathway mutations |
| BRSK2 | 0.004 | 0.053 | 13.500 | 2.74E-30 | 1.15E-29 | 28.940 | 3.755 | Upregulated DNA damage pathway mutations |
| BUB1B | 0.006 | 0.060 | 10.629 | 7.01E-31 | 3.08E-30 | 29.512 | 3.410 | Upregulated DNA damage pathway mutations |
| BUB3 | 0.003 | 0.022 | 7.697 | 3.11E-10 | 4.01E-10 | 9.397 | 2.944 | Upregulated DNA damage pathway mutations |
| CABIN1 | 0.013 | 0.105 | 7.967 | 3.75E-46 | 8.92E-45 | 44.050 | 2.994 | Upregulated DNA damage pathway mutations |
| CAD | 0.018 | 0.118 | 6.466 | 3.73E-45 | 8.19E-44 | 43.087 | 2.693 | Upregulated DNA damage pathway mutations |
| CCAR2 | 0.007 | 0.068 | 9.827 | 1.10E-33 | 6.79E-33 | 32.168 | 3.297 | Upregulated DNA damage pathway mutations |
| CCNA2 | 0.002 | 0.030 | 12.015 | 6.26E-17 | 1.15E-16 | 15.939 | 3.587 | Upregulated DNA damage pathway mutations |
| CCNB1 | 0.002 | 0.026 | 11.912 | 1.02E-14 | 1.60E-14 | 13.796 | 3.574 | Upregulated DNA damage pathway mutations |
| CCND1 | 0.004 | 0.028 | 6.276 | 2.63E-11 | 3.44E-11 | 10.463 | 2.650 | Upregulated DNA damage pathway mutations |
| CCND2 | 0.002 | 0.029 | 12.128 | 1.87E-16 | 3.31E-16 | 15.480 | 3.600 | Upregulated DNA damage pathway mutations |
| CCND3 | 0.002 | 0.012 | 6.353 | 8.62E-06 | 9.20E-06 | 5.036 | 2.667 | Upregulated DNA damage pathway mutations |
| CCNE1 | 0.004 | 0.036 | 9.810 | 2.18E-18 | 4.40E-18 | 17.356 | 3.294 | Upregulated DNA damage pathway mutations |
| CCNE2 | 0.004 | 0.025 | 5.578 | 1.94E-09 | 2.36E-09 | 8.627 | 2.480 | Upregulated DNA damage pathway mutations |
| CCNF | 0.006 | 0.042 | 7.235 | 4.76E-18 | 9.22E-18 | 17.035 | 2.855 | Upregulated DNA damage pathway mutations |
| CCNH | 0.002 | 0.020 | 9.053 | 3.45E-10 | 4.41E-10 | 9.355 | 3.178 | Upregulated DNA damage pathway mutations |
| CCP110 | 0.006 | 0.060 | 9.697 | 1.62E-29 | 6.14E-29 | 28.212 | 3.277 | Upregulated DNA damage pathway mutations |
| CDC16 | 0.003 | 0.052 | 16.430 | 5.28E-32 | 2.60E-31 | 30.586 | 4.038 | Upregulated DNA damage pathway mutations |
| CDC20 | 0.004 | 0.024 | 5.479 | 5.46E-09 | 6.51E-09 | 8.187 | 2.454 | Upregulated DNA damage pathway mutations |
| CDC23 | 0.003 | 0.039 | 12.487 | 3.78E-22 | 9.46E-22 | 21.024 | 3.642 | Upregulated DNA damage pathway mutations |
| CDC25A | 0.003 | 0.038 | 11.018 | 1.87E-20 | 4.22E-20 | 19.375 | 3.462 | Upregulated DNA damage pathway mutations |
| CDC25B | 0.004 | 0.034 | 8.276 | 5.91E-16 | 1.01E-15 | 14.996 | 3.049 | Upregulated DNA damage pathway mutations |
| CDC25C | 0.003 | 0.047 | 14.294 | 1.82E-27 | 6.41E-27 | 26.193 | 3.837 | Upregulated DNA damage pathway mutations |
| CDC27 | 0.006 | 0.061 | 10.040 | 1.54E-30 | 6.57E-30 | 29.182 | 3.328 | Upregulated DNA damage pathway mutations |
| CDC37 | 0.003 | 0.027 | 8.544 | 4.56E-13 | 6.47E-13 | 12.189 | 3.095 | Upregulated DNA damage pathway mutations |
| CDC45 | 0.004 | 0.049 | 11.484 | 3.83E-26 | 1.17E-25 | 24.930 | 3.522 | Upregulated DNA damage pathway mutations |
| CDC6 | 0.005 | 0.024 | 4.871 | 2.93E-08 | 3.38E-08 | 7.471 | 2.284 | Upregulated DNA damage pathway mutations |
| CDC7 | 0.006 | 0.052 | 9.343 | 4.09E-25 | 1.19E-24 | 23.925 | 3.224 | Upregulated DNA damage pathway mutations |
| CDC73 | 0.007 | 0.056 | 8.168 | 3.93E-25 | 1.15E-24 | 23.938 | 3.030 | Upregulated DNA damage pathway mutations |
| CDCA2 | 0.009 | 0.060 | 6.659 | 9.64E-24 | 2.54E-23 | 22.594 | 2.735 | Upregulated DNA damage pathway mutations |
| CDIP1 | 0.001 | 0.007 | 13.341 | 3.52E-05 | 3.70E-05 | 4.431 | 3.738 | Upregulated DNA damage pathway mutations |
| CDK1 | 0.002 | 0.024 | 12.177 | 9.08E-14 | 1.34E-13 | 12.873 | 3.606 | Upregulated DNA damage pathway mutations |
| CDK10 | 0.003 | 0.020 | 6.243 | 2.52E-08 | 2.92E-08 | 7.535 | 2.642 | Upregulated DNA damage pathway mutations |
| CDK11B | 0.005 | 0.035 | 6.750 | 1.48E-14 | 2.31E-14 | 13.636 | 2.755 | Upregulated DNA damage pathway mutations |
| CDK12 | 0.019 | 0.097 | 5.119 | 3.15E-31 | 1.43E-30 | 29.846 | 2.356 | Upregulated DNA damage pathway mutations |
| CDK16 | 0.002 | 0.034 | 14.975 | 8.06E-21 | 1.84E-20 | 19.736 | 3.904 | Upregulated DNA damage pathway mutations |
| CDK2 | 0.002 | 0.018 | 10.125 | 9.76E-10 | 1.23E-09 | 8.912 | 3.340 | Upregulated DNA damage pathway mutations |
| CDK4 | 0.002 | 0.024 | 9.963 | 1.27E-12 | 1.76E-12 | 11.754 | 3.317 | Upregulated DNA damage pathway mutations |
| CDK5 | 0.002 | 0.023 | 9.115 | 1.25E-11 | 1.66E-11 | 10.780 | 3.188 | Upregulated DNA damage pathway mutations |
| CDK6 | 0.002 | 0.027 | 12.388 | 1.67E-15 | 2.78E-15 | 14.557 | 3.631 | Upregulated DNA damage pathway mutations |
| CDK7 | 0.003 | 0.027 | 9.911 | 4.62E-14 | 6.96E-14 | 13.157 | 3.309 | Upregulated DNA damage pathway mutations |
| CDK8 | 0.005 | 0.039 | 7.705 | 1.85E-17 | 3.50E-17 | 16.456 | 2.946 | Upregulated DNA damage pathway mutations |
| CDK9 | 0.003 | 0.022 | 8.338 | 1.13E-10 | 1.46E-10 | 9.836 | 3.060 | Upregulated DNA damage pathway mutations |
| CDKN1A | 0.007 | 0.031 | 4.332 | 2.02E-09 | 2.44E-09 | 8.612 | 2.115 | Upregulated DNA damage pathway mutations |
| CDKN1B | 0.005 | 0.024 | 5.219 | 1.09E-08 | 1.28E-08 | 7.893 | 2.384 | Upregulated DNA damage pathway mutations |
| CDKN1C | 0.000 | 0.003 | 7.147 | 0.022 | 2.25E-02 | 1.648 | 2.837 | Upregulated DNA damage pathway mutations |
| CDKN2A | 0.035 | 0.057 | 1.613 | 0.002 | 0.002 | 2.763 | 0.689 | Upregulated DNA damage pathway mutations |
| CDKN2C | 0.002 | 0.016 | 6.497 | 5.22E-07 | 5.79E-07 | 6.237 | 2.700 | Upregulated DNA damage pathway mutations |
| CDT1 | 0.002 | 0.033 | 13.258 | 2.60E-19 | 5.58E-19 | 18.254 | 3.729 | Upregulated DNA damage pathway mutations |
| CEND1 | 0.001 | 0.008 | 8.471 | 7.16E-05 | 7.44E-05 | 4.128 | 3.082 | Upregulated DNA damage pathway mutations |
| CENPA | 0.001 | 0.011 | 13.103 | 1.98E-07 | 2.23E-07 | 6.651 | 3.712 | Upregulated DNA damage pathway mutations |
| CENPE | 0.023 | 0.117 | 4.985 | 1.10E-36 | 1.01E-35 | 34.996 | 2.318 | Upregulated DNA damage pathway mutations |
| CENPF | 0.024 | 0.137 | 5.666 | 1.37E-47 | 3.90E-46 | 45.409 | 2.502 | Upregulated DNA damage pathway mutations |
| CENPI | 0.005 | 0.063 | 12.918 | 2.20E-35 | 1.57E-34 | 33.804 | 3.691 | Upregulated DNA damage pathway mutations |
| CENPT | 0.003 | 0.030 | 11.054 | 2.48E-16 | 4.33E-16 | 15.363 | 3.467 | Upregulated DNA damage pathway mutations |
| CEP55 | 0.004 | 0.040 | 11.262 | 1.05E-21 | 2.50E-21 | 20.601 | 3.493 | Upregulated DNA damage pathway mutations |
| CHEK1 | 0.005 | 0.036 | 7.941 | 1.84E-16 | 3.27E-16 | 15.485 | 2.989 | Upregulated DNA damage pathway mutations |
| CHEK2 | 0.008 | 0.041 | 5.222 | 4.02E-14 | 6.12E-14 | 13.213 | 2.384 | Upregulated DNA damage pathway mutations |
| CHFR | 0.005 | 0.049 | 10.416 | 5.46E-25 | 1.57E-24 | 23.803 | 3.381 | Upregulated DNA damage pathway mutations |
| CIRBP | 0.002 | 0.011 | 5.517 | 5.36E-05 | 5.62E-05 | 4.250 | 2.464 | Upregulated DNA damage pathway mutations |
| CKAP5 | 0.015 | 0.090 | 5.880 | 3.10E-32 | 1.58E-31 | 30.801 | 2.556 | Upregulated DNA damage pathway mutations |
| CLASP2 | 0.010 | 0.081 | 8.447 | 1.38E-36 | 1.23E-35 | 34.910 | 3.078 | Upregulated DNA damage pathway mutations |
| CLSPN | 0.010 | 0.080 | 8.245 | 1.25E-35 | 9.34E-35 | 34.029 | 3.043 | Upregulated DNA damage pathway mutations |
| COPS5 | 0.003 | 0.021 | 7.624 | 9.29E-10 | 1.17E-09 | 8.931 | 2.930 | Upregulated DNA damage pathway mutations |
| CSNK1A1 | 0.003 | 0.027 | 10.324 | 2.48E-14 | 3.85E-14 | 13.415 | 3.368 | Upregulated DNA damage pathway mutations |
| CSNK2A1 | 0.005 | 0.035 | 7.200 | 3.82E-15 | 6.26E-15 | 14.204 | 2.848 | Upregulated DNA damage pathway mutations |
| CUEDC2 | 0.002 | 0.024 | 10.959 | 3.58E-13 | 5.13E-13 | 12.290 | 3.454 | Upregulated DNA damage pathway mutations |
| DCLRE1C | 0.006 | 0.044 | 8.035 | 4.57E-20 | 1.01E-19 | 18.996 | 3.006 | Upregulated DNA damage pathway mutations |
| DCTN1 | 0.010 | 0.089 | 8.627 | 9.16E-41 | 1.37E-39 | 38.862 | 3.109 | Upregulated DNA damage pathway mutations |
| DDB1 | 0.008 | 0.078 | 9.658 | 7.72E-38 | 8.46E-37 | 36.072 | 3.272 | Upregulated DNA damage pathway mutations |
| DDB2 | 0.003 | 0.030 | 10.629 | 4.77E-16 | 8.20E-16 | 15.086 | 3.410 | Upregulated DNA damage pathway mutations |
| DDIT4 | 0.002 | 0.017 | 8.471 | 1.65E-08 | 1.92E-08 | 7.717 | 3.082 | Upregulated DNA damage pathway mutations |
| DDX3X | 0.012 | 0.077 | 6.590 | 8.09E-30 | 3.16E-29 | 28.500 | 2.720 | Upregulated DNA damage pathway mutations |
| DMAP1 | 0.003 | 0.030 | 8.915 | 9.61E-15 | 1.52E-14 | 13.817 | 3.156 | Upregulated DNA damage pathway mutations |
| DPYD | 0.017 | 0.132 | 7.819 | 2.12E-57 | 1.21E-55 | 54.917 | 2.967 | Upregulated DNA damage pathway mutations |
| DTX3L | 0.005 | 0.043 | 9.308 | 3.02E-21 | 7.06E-21 | 20.151 | 3.218 | Upregulated DNA damage pathway mutations |
| DYRK2 | 0.005 | 0.040 | 8.643 | 4.58E-19 | 9.52E-19 | 18.021 | 3.112 | Upregulated DNA damage pathway mutations |
| E2F1 | 0.003 | 0.035 | 10.125 | 3.57E-18 | 7.06E-18 | 17.151 | 3.340 | Upregulated DNA damage pathway mutations |
| EAPP | 0.003 | 0.026 | 8.508 | 1.37E-12 | 1.89E-12 | 11.723 | 3.089 | Upregulated DNA damage pathway mutations |
| ENSA | 0.001 | 0.014 | 16.677 | 6.86E-10 | 8.69E-10 | 9.061 | 4.060 | Upregulated DNA damage pathway mutations |
| ERCC1 | 0.002 | 0.031 | 12.430 | 1.02E-17 | 1.94E-17 | 16.712 | 3.636 | Upregulated DNA damage pathway mutations |
| ERCC2 | 0.007 | 0.046 | 6.077 | 3.28E-17 | 6.11E-17 | 16.214 | 2.603 | Upregulated DNA damage pathway mutations |
| ERCC3 | 0.006 | 0.061 | 10.223 | 8.25E-31 | 3.56E-30 | 29.448 | 3.354 | Upregulated DNA damage pathway mutations |
| ERCC4 | 0.007 | 0.074 | 10.090 | 3.64E-37 | 3.70E-36 | 35.432 | 3.335 | Upregulated DNA damage pathway mutations |
| ERCC6L | 0.009 | 0.063 | 7.266 | 2.38E-26 | 7.55E-26 | 25.122 | 2.861 | Upregulated DNA damage pathway mutations |
| FANCA | 0.010 | 0.081 | 7.992 | 1.93E-35 | 1.41E-34 | 33.850 | 2.999 | Upregulated DNA damage pathway mutations |
| FANCB | 0.007 | 0.069 | 9.389 | 2.15E-33 | 1.31E-32 | 31.884 | 3.231 | Upregulated DNA damage pathway mutations |
| FANCD2 | 0.010 | 0.084 | 8.212 | 2.31E-37 | 2.43E-36 | 35.614 | 3.038 | Upregulated DNA damage pathway mutations |
| FEN1 | 0.002 | 0.023 | 11.034 | 1.08E-12 | 1.51E-12 | 11.822 | 3.464 | Upregulated DNA damage pathway mutations |
| FOS | 0.003 | 0.029 | 9.882 | 5.05E-15 | 8.18E-15 | 14.087 | 3.305 | Upregulated DNA damage pathway mutations |
| FOSB | 0.003 | 0.026 | 7.685 | 6.41E-12 | 8.57E-12 | 11.067 | 2.942 | Upregulated DNA damage pathway mutations |
| FOXK2 | 0.004 | 0.031 | 7.727 | 4.42E-14 | 6.70E-14 | 13.174 | 2.950 | Upregulated DNA damage pathway mutations |
| FOXM1 | 0.007 | 0.060 | 8.503 | 1.57E-27 | 5.59E-27 | 26.252 | 3.088 | Upregulated DNA damage pathway mutations |
| GADD45A | 0.001 | 0.004 | 4.765 | 0.022 | 2.17E-02 | 1.663 | 2.252 | Upregulated DNA damage pathway mutations |
| GMNN | 0.002 | 0.021 | 12.706 | 2.40E-12 | 3.28E-12 | 11.484 | 3.667 | Upregulated DNA damage pathway mutations |
| GSTP1 | 0.001 | 0.011 | 8.063 | 4.18E-06 | 4.55E-06 | 5.342 | 3.011 | Upregulated DNA damage pathway mutations |
| HCFC1 | 0.015 | 0.105 | 7.200 | 2.28E-43 | 3.82E-42 | 41.418 | 2.848 | Upregulated DNA damage pathway mutations |
| HELQ | 0.009 | 0.070 | 8.100 | 3.09E-31 | 1.42E-30 | 29.848 | 3.018 | Upregulated DNA damage pathway mutations |
| HIPK2 | 0.010 | 0.059 | 6.172 | 3.51E-22 | 8.84E-22 | 21.053 | 2.626 | Upregulated DNA damage pathway mutations |
| HJURP | 0.007 | 0.051 | 6.969 | 7.75E-21 | 1.78E-20 | 19.749 | 2.801 | Upregulated DNA damage pathway mutations |
| HUS1 | 0.002 | 0.016 | 6.497 | 5.22E-07 | 5.79E-07 | 6.237 | 2.700 | Upregulated DNA damage pathway mutations |
| INCENP | 0.006 | 0.055 | 8.861 | 8.01E-26 | 2.38E-25 | 24.624 | 3.147 | Upregulated DNA damage pathway mutations |
| JUNB | 0.002 | 0.012 | 6.727 | 5.66E-06 | 6.13E-06 | 5.212 | 2.750 | Upregulated DNA damage pathway mutations |
| KAT5 | 0.004 | 0.038 | 9.041 | 1.38E-18 | 2.83E-18 | 17.549 | 3.176 | Upregulated DNA damage pathway mutations |
| KIF11 | 0.004 | 0.071 | 16.438 | 7.86E-44 | 1.49E-42 | 41.826 | 4.039 | Upregulated DNA damage pathway mutations |
| LATS1 | 0.013 | 0.093 | 7.268 | 1.61E-38 | 2.00E-37 | 36.699 | 2.862 | Upregulated DNA damage pathway mutations |
| LATS2 | 0.008 | 0.074 | 9.529 | 4.21E-36 | 3.42E-35 | 34.465 | 3.252 | Upregulated DNA damage pathway mutations |
| LIG4 | 0.010 | 0.069 | 7.016 | 3.26E-28 | 1.18E-27 | 26.930 | 2.811 | Upregulated DNA damage pathway mutations |
| LMNA | 0.005 | 0.031 | 6.083 | 3.68E-12 | 4.99E-12 | 11.302 | 2.605 | Upregulated DNA damage pathway mutations |
| MAD2L1 | 0.002 | 0.024 | 14.612 | 9.19E-15 | 1.46E-14 | 13.835 | 3.869 | Upregulated DNA damage pathway mutations |
| MAPRE1 | 0.002 | 0.024 | 11.536 | 1.83E-13 | 2.67E-13 | 12.573 | 3.528 | Upregulated DNA damage pathway mutations |
| MASTL | 0.007 | 0.066 | 9.837 | 1.04E-32 | 5.82E-32 | 31.235 | 3.298 | Upregulated DNA damage pathway mutations |
| MCM2 | 0.006 | 0.056 | 9.356 | 4.69E-27 | 1.61E-26 | 25.793 | 3.226 | Upregulated DNA damage pathway mutations |
| MCM3 | 0.006 | 0.040 | 6.637 | 2.51E-16 | 4.35E-16 | 15.361 | 2.730 | Upregulated DNA damage pathway mutations |
| MCM4 | 0.009 | 0.065 | 7.147 | 6.70E-27 | 2.27E-26 | 25.643 | 2.837 | Upregulated DNA damage pathway mutations |
| MCM7 | 0.008 | 0.049 | 6.308 | 8.96E-19 | 1.85E-18 | 17.733 | 2.657 | Upregulated DNA damage pathway mutations |
| MCPH1 | 0.007 | 0.067 | 9.529 | 1.13E-32 | 6.09E-32 | 31.215 | 3.252 | Upregulated DNA damage pathway mutations |
| MDM2 | 0.004 | 0.023 | 5.666 | 7.43E-09 | 8.78E-09 | 8.056 | 2.502 | Upregulated DNA damage pathway mutations |
| MEX3C | 0.003 | 0.030 | 9.212 | 5.45E-15 | 8.78E-15 | 14.056 | 3.203 | Upregulated DNA damage pathway mutations |
| MGMT | 0.002 | 0.025 | 13.453 | 6.93E-15 | 1.11E-14 | 13.955 | 3.750 | Upregulated DNA damage pathway mutations |
| MIS18A | 0.001 | 0.010 | 8.663 | 7.58E-06 | 8.12E-06 | 5.091 | 3.115 | Upregulated DNA damage pathway mutations |
| MKI67 | 0.032 | 0.170 | 5.316 | 1.27E-56 | 5.18E-55 | 54.286 | 2.410 | Upregulated DNA damage pathway mutations |
| MLH1 | 0.007 | 0.073 | 11.277 | 1.15E-38 | 1.49E-37 | 36.827 | 3.495 | Upregulated DNA damage pathway mutations |
| MSH2 | 0.008 | 0.074 | 9.399 | 7.62E-36 | 6.03E-35 | 34.220 | 3.232 | Upregulated DNA damage pathway mutations |
| MSH6 | 0.009 | 0.105 | 11.854 | 1.12E-56 | 5.18E-55 | 54.286 | 3.567 | Upregulated DNA damage pathway mutations |
| MUTYH | 0.005 | 0.041 | 8.865 | 8.70E-20 | 1.91E-19 | 18.719 | 3.148 | Upregulated DNA damage pathway mutations |
| NAA10 | 0.003 | 0.017 | 5.082 | 2.47E-06 | 2.71E-06 | 5.567 | 2.346 | Upregulated DNA damage pathway mutations |
| NBN | 0.008 | 0.066 | 8.355 | 5.61E-30 | 2.22E-29 | 28.654 | 3.063 | Upregulated DNA damage pathway mutations |
| NCAPD3 | 0.010 | 0.107 | 10.669 | 1.71E-54 | 6.09E-53 | 52.215 | 3.415 | Upregulated DNA damage pathway mutations |
| NEK7 | 0.002 | 0.033 | 13.861 | 1.19E-19 | 2.60E-19 | 18.585 | 3.793 | Upregulated DNA damage pathway mutations |
| NHEJ1 | 0.001 | 0.022 | 15.394 | 7.69E-14 | 1.15E-13 | 12.940 | 3.944 | Upregulated DNA damage pathway mutations |
| NPM1 | 0.003 | 0.018 | 5.586 | 4.43E-07 | 4.95E-07 | 6.305 | 2.482 | Upregulated DNA damage pathway mutations |
| NTMT1 | 0.002 | 0.024 | 13.699 | 2.04E-14 | 3.18E-14 | 13.498 | 3.776 | Upregulated DNA damage pathway mutations |
| NUDT1 | 0.002 | 0.009 | 4.288 | 0.001 | 0.001 | 2.965 | 2.100 | Upregulated DNA damage pathway mutations |
| NUMA1 | 0.015 | 0.112 | 7.351 | 1.56E-46 | 4.04E-45 | 44.394 | 2.878 | Upregulated DNA damage pathway mutations |
| ORC1 | 0.007 | 0.062 | 9.222 | 1.01E-29 | 3.91E-29 | 28.408 | 3.205 | Upregulated DNA damage pathway mutations |
| ORC2 | 0.005 | 0.039 | 8.047 | 6.77E-18 | 1.30E-17 | 16.885 | 3.008 | Upregulated DNA damage pathway mutations |
| ORC6 | 0.002 | 0.012 | 5.718 | 1.88E-05 | 1.99E-05 | 4.702 | 2.515 | Upregulated DNA damage pathway mutations |
| OTUB1 | 0.002 | 0.017 | 10.891 | 1.57E-09 | 1.94E-09 | 8.713 | 3.445 | Upregulated DNA damage pathway mutations |
| PARG | 0.006 | 0.061 | 10.812 | 1.19E-31 | 5.56E-31 | 30.255 | 3.435 | Upregulated DNA damage pathway mutations |
| PBK | 0.002 | 0.021 | 11.912 | 4.93E-12 | 6.63E-12 | 11.179 | 3.574 | Upregulated DNA damage pathway mutations |
| PCM1 | 0.009 | 0.075 | 7.996 | 3.19E-33 | 1.89E-32 | 31.723 | 2.999 | Upregulated DNA damage pathway mutations |
| PCNA | 0.002 | 0.018 | 8.100 | 9.21E-09 | 1.08E-08 | 7.967 | 3.018 | Upregulated DNA damage pathway mutations |
| PHB | 0.002 | 0.020 | 8.230 | 1.02E-09 | 1.27E-09 | 8.897 | 3.041 | Upregulated DNA damage pathway mutations |
| PHLDA3 | 0.002 | 0.003 | 1.588 | 0.444 | 4.44E-01 | 0.352 | 0.667 | Non-significant |
| PIN1 | 0.001 | 0.010 | 10.588 | 2.37E-06 | 2.61E-06 | 5.584 | 3.404 | Upregulated DNA damage pathway mutations |
| PKMYT1 | 0.002 | 0.027 | 15.485 | 7.18E-17 | 1.31E-16 | 15.882 | 3.953 | Upregulated DNA damage pathway mutations |
| PLK1 | 0.006 | 0.049 | 7.998 | 9.20E-22 | 2.22E-21 | 20.653 | 3.000 | Upregulated DNA damage pathway mutations |
| PLK2 | 0.005 | 0.053 | 9.720 | 4.07E-26 | 1.24E-25 | 24.908 | 3.281 | Upregulated DNA damage pathway mutations |
| PLK3 | 0.004 | 0.043 | 10.006 | 4.99E-22 | 1.23E-21 | 20.911 | 3.323 | Upregulated DNA damage pathway mutations |
| PNKP | 0.003 | 0.026 | 9.163 | 4.54E-13 | 6.47E-13 | 12.189 | 3.196 | Upregulated DNA damage pathway mutations |
| POLG | 0.008 | 0.056 | 7.049 | 5.21E-23 | 1.35E-22 | 21.870 | 2.817 | Upregulated DNA damage pathway mutations |
| POLH | 0.003 | 0.051 | 14.592 | 4.79E-30 | 1.92E-29 | 28.716 | 3.867 | Upregulated DNA damage pathway mutations |
| POLR2A | 0.013 | 0.099 | 7.753 | 9.75E-43 | 1.54E-41 | 40.811 | 2.955 | Upregulated DNA damage pathway mutations |
| PPM1D | 0.005 | 0.053 | 10.125 | 1.19E-26 | 3.86E-26 | 25.414 | 3.340 | Upregulated DNA damage pathway mutations |
| PPP1CA | 0.002 | 0.025 | 10.396 | 2.26E-13 | 3.29E-13 | 12.482 | 3.378 | Upregulated DNA damage pathway mutations |
| PPP2CA | 0.003 | 0.025 | 8.168 | 7.00E-12 | 9.32E-12 | 11.031 | 3.030 | Upregulated DNA damage pathway mutations |
| PPP2R1A | 0.015 | 0.059 | 3.936 | 2.87E-15 | 4.76E-15 | 14.322 | 1.977 | Upregulated DNA damage pathway mutations |
| PPP2R2A | 0.005 | 0.040 | 8.849 | 2.64E-19 | 5.61E-19 | 18.251 | 3.145 | Upregulated DNA damage pathway mutations |
| PPP5C | 0.004 | 0.037 | 8.367 | 2.16E-17 | 4.05E-17 | 16.392 | 3.065 | Upregulated DNA damage pathway mutations |
| PRIM1 | 0.003 | 0.019 | 6.597 | 3.19E-08 | 3.66E-08 | 7.436 | 2.722 | Upregulated DNA damage pathway mutations |
| PRIM2 | 0.002 | 0.019 | 12.252 | 4.42E-11 | 5.75E-11 | 10.240 | 3.615 | Upregulated DNA damage pathway mutations |
| PRKDC | 0.034 | 0.185 | 5.432 | 3.78E-63 | 5.39E-61 | 60.268 | 2.442 | Upregulated DNA damage pathway mutations |
| PTPN12 | 0.007 | 0.064 | 8.952 | 3.38E-30 | 1.40E-29 | 28.855 | 3.162 | Upregulated DNA damage pathway mutations |
| PTPRA | 0.007 | 0.061 | 8.785 | 1.71E-28 | 6.34E-28 | 27.198 | 3.135 | Upregulated DNA damage pathway mutations |
| PTTG1 | 0.001 | 0.013 | 10.324 | 8.30E-08 | 9.42E-08 | 7.026 | 3.368 | Upregulated DNA damage pathway mutations |
| RAD17 | 0.005 | 0.050 | 10.891 | 4.89E-26 | 1.47E-25 | 24.834 | 3.445 | Upregulated DNA damage pathway mutations |
| RAD18 | 0.004 | 0.049 | 11.786 | 1.91E-26 | 6.11E-26 | 25.214 | 3.559 | Upregulated DNA damage pathway mutations |
| RAD21 | 0.007 | 0.049 | 6.491 | 3.89E-19 | 8.21E-19 | 18.086 | 2.698 | Upregulated DNA damage pathway mutations |
| RAD23A | 0.004 | 0.026 | 7.219 | 1.66E-11 | 2.20E-11 | 10.658 | 2.852 | Upregulated DNA damage pathway mutations |
| RAD23B | 0.003 | 0.033 | 11.294 | 4.64E-18 | 9.06E-18 | 17.043 | 3.498 | Upregulated DNA damage pathway mutations |
| RAD50 | 0.010 | 0.086 | 8.505 | 4.79E-39 | 6.51E-38 | 37.187 | 3.088 | Upregulated DNA damage pathway mutations |
| RAD51 | 0.002 | 0.020 | 8.230 | 1.02E-09 | 1.27E-09 | 8.897 | 3.041 | Upregulated DNA damage pathway mutations |
| RAD54L | 0.005 | 0.049 | 10.179 | 1.03E-24 | 2.90E-24 | 23.537 | 3.348 | Upregulated DNA damage pathway mutations |
| RAD9A | 0.002 | 0.019 | 7.458 | 8.25E-09 | 9.72E-09 | 8.012 | 2.899 | Upregulated DNA damage pathway mutations |
| RB1 | 0.037 | 0.102 | 2.742 | 1.50E-16 | 2.70E-16 | 15.569 | 1.455 | Upregulated DNA damage pathway mutations |
| RBBP8 | 0.006 | 0.061 | 10.412 | 4.37E-31 | 1.95E-30 | 29.711 | 3.380 | Upregulated DNA damage pathway mutations |
| RBL2 | 0.008 | 0.073 | 8.787 | 4.17E-34 | 2.70E-33 | 32.569 | 3.135 | Upregulated DNA damage pathway mutations |
| RBMX | 0.004 | 0.043 | 10.817 | 7.38E-23 | 1.89E-22 | 21.723 | 3.435 | Upregulated DNA damage pathway mutations |
| RCC1 | 0.002 | 0.030 | 12.015 | 6.26E-17 | 1.15E-16 | 15.939 | 3.587 | Upregulated DNA damage pathway mutations |
| RCC2 | 0.004 | 0.041 | 10.891 | 6.76E-22 | 1.65E-21 | 20.783 | 3.445 | Upregulated DNA damage pathway mutations |
| RCHY1 | 0.002 | 0.022 | 12.507 | 8.07E-13 | 1.13E-12 | 11.946 | 3.645 | Upregulated DNA damage pathway mutations |
| RECQL | 0.005 | 0.044 | 9.106 | 1.77E-21 | 4.20E-21 | 20.376 | 3.187 | Upregulated DNA damage pathway mutations |
| RECQL4 | 0.007 | 0.056 | 8.040 | 6.59E-25 | 1.88E-24 | 23.726 | 3.007 | Upregulated DNA damage pathway mutations |
| RECQL5 | 0.008 | 0.061 | 7.919 | 7.10E-27 | 2.37E-26 | 25.625 | 2.985 | Upregulated DNA damage pathway mutations |
| RIF1 | 0.018 | 0.140 | 7.658 | 7.61E-60 | 5.42E-58 | 57.266 | 2.937 | Upregulated DNA damage pathway mutations |
| RNF2 | 0.002 | 0.026 | 12.539 | 4.96E-15 | 8.07E-15 | 14.093 | 3.648 | Upregulated DNA damage pathway mutations |
| RPA1 | 0.004 | 0.029 | 7.848 | 2.40E-13 | 3.48E-13 | 12.459 | 2.972 | Upregulated DNA damage pathway mutations |
| RPA2 | 0.002 | 0.016 | 7.941 | 8.33E-08 | 9.42E-08 | 7.026 | 2.989 | Upregulated DNA damage pathway mutations |
| RRM1 | 0.004 | 0.039 | 9.285 | 2.56E-19 | 5.53E-19 | 18.257 | 3.215 | Upregulated DNA damage pathway mutations |
| S100A6 | 0.000 | 0.004 | 9.529 | 0.004 | 0.004 | 2.375 | 3.252 | Upregulated DNA damage pathway mutations |
| SESN2 | 0.004 | 0.043 | 11.772 | 9.59E-24 | 2.54E-23 | 22.594 | 3.557 | Upregulated DNA damage pathway mutations |
| SFN | 0.002 | 0.011 | 7.487 | 6.83E-06 | 7.38E-06 | 5.132 | 2.904 | Upregulated DNA damage pathway mutations |
| SKP2 | 0.003 | 0.035 | 12.960 | 2.97E-20 | 6.61E-20 | 19.180 | 3.696 | Upregulated DNA damage pathway mutations |
| SLK | 0.009 | 0.073 | 8.353 | 3.71E-33 | 2.15E-32 | 31.667 | 3.062 | Upregulated DNA damage pathway mutations |
| SMARCA4 | 0.025 | 0.134 | 5.317 | 1.42E-44 | 2.89E-43 | 42.538 | 2.411 | Upregulated DNA damage pathway mutations |
| SMARCA5 | 0.006 | 0.059 | 10.446 | 4.10E-30 | 1.67E-29 | 28.777 | 3.385 | Upregulated DNA damage pathway mutations |
| SMARCAL1 | 0.008 | 0.079 | 10.201 | 2.09E-39 | 2.98E-38 | 37.525 | 3.351 | Upregulated DNA damage pathway mutations |
| SMC1A | 0.012 | 0.082 | 6.907 | 8.72E-33 | 4.97E-32 | 31.304 | 2.788 | Upregulated DNA damage pathway mutations |
| SMC2 | 0.012 | 0.086 | 7.462 | 3.80E-36 | 3.19E-35 | 34.496 | 2.900 | Upregulated DNA damage pathway mutations |
| SMC3 | 0.011 | 0.069 | 6.515 | 7.16E-27 | 2.37E-26 | 25.625 | 2.704 | Upregulated DNA damage pathway mutations |
| SMC4 | 0.012 | 0.087 | 7.022 | 3.38E-35 | 2.35E-34 | 33.629 | 2.812 | Upregulated DNA damage pathway mutations |
| SMG1 | 0.022 | 0.126 | 5.616 | 1.24E-43 | 2.21E-42 | 41.655 | 2.490 | Upregulated DNA damage pathway mutations |
| STAG2 | 0.019 | 0.098 | 5.203 | 5.59E-32 | 2.70E-31 | 30.569 | 2.379 | Upregulated DNA damage pathway mutations |
| TACC3 | 0.007 | 0.053 | 7.839 | 3.05E-23 | 7.97E-23 | 22.099 | 2.971 | Upregulated DNA damage pathway mutations |
| TDP1 | 0.006 | 0.034 | 6.048 | 3.42E-13 | 4.93E-13 | 12.307 | 2.596 | Upregulated DNA damage pathway mutations |
| TERF2 | 0.003 | 0.032 | 12.309 | 3.43E-18 | 6.83E-18 | 17.166 | 3.622 | Upregulated DNA damage pathway mutations |
| TERF2IP | 0.002 | 0.020 | 9.053 | 3.45E-10 | 4.41E-10 | 9.355 | 3.178 | Upregulated DNA damage pathway mutations |
| TFAP2C | 0.005 | 0.036 | 6.949 | 3.22E-15 | 5.31E-15 | 14.275 | 2.797 | Upregulated DNA damage pathway mutations |
| TFCP2 | 0.003 | 0.056 | 18.378 | 7.91E-36 | 6.09E-35 | 34.215 | 4.200 | Upregulated DNA damage pathway mutations |
| TLK1 | 0.005 | 0.063 | 11.863 | 4.15E-34 | 2.70E-33 | 32.569 | 3.568 | Upregulated DNA damage pathway mutations |
| TMPO | 0.007 | 0.066 | 9.529 | 3.49E-32 | 1.75E-31 | 30.758 | 3.252 | Upregulated DNA damage pathway mutations |
| TOP1 | 0.006 | 0.068 | 12.095 | 3.78E-37 | 3.71E-36 | 35.430 | 3.596 | Upregulated DNA damage pathway mutations |
| TOP2A | 0.011 | 0.084 | 7.351 | 5.79E-35 | 3.93E-34 | 33.406 | 2.878 | Upregulated DNA damage pathway mutations |
| TOPBP1 | 0.010 | 0.078 | 7.445 | 1.11E-32 | 6.09E-32 | 31.215 | 2.896 | Upregulated DNA damage pathway mutations |
| TP53 | 0.362 | 0.384 | 1.060 | 0.182 | 1.83E-01 | 0.738 | 0.084 | Non-significant |
| TP53BP1 | 0.018 | 0.095 | 5.346 | 1.15E-31 | 5.48E-31 | 30.261 | 2.418 | Upregulated DNA damage pathway mutations |
| TP63 | 0.016 | 0.097 | 6.221 | 3.07E-36 | 2.65E-35 | 34.577 | 2.637 | Upregulated DNA damage pathway mutations |
| TP73 | 0.006 | 0.042 | 7.104 | 7.56E-18 | 1.45E-17 | 16.840 | 2.829 | Upregulated DNA damage pathway mutations |
| TPT1 | 0.001 | 0.009 | 10.721 | 7.29E-06 | 7.84E-06 | 5.106 | 3.422 | Upregulated DNA damage pathway mutations |
| TPX2 | 0.007 | 0.042 | 5.831 | 1.14E-15 | 1.92E-15 | 14.717 | 2.544 | Upregulated DNA damage pathway mutations |
| TRIB2 | 0.003 | 0.040 | 15.485 | 1.10E-24 | 3.07E-24 | 23.513 | 3.953 | Upregulated DNA damage pathway mutations |
| TRIM28 | 0.006 | 0.043 | 7.277 | 1.60E-18 | 3.25E-18 | 17.488 | 2.863 | Upregulated DNA damage pathway mutations |
| TRIM29 | 0.007 | 0.059 | 8.357 | 8.21E-27 | 2.69E-26 | 25.571 | 3.063 | Upregulated DNA damage pathway mutations |
| TRRAP | 0.034 | 0.188 | 5.541 | 2.85E-65 | 8.11E-63 | 62.091 | 2.470 | Upregulated DNA damage pathway mutations |
| TTK | 0.012 | 0.089 | 7.659 | 4.88E-38 | 5.56E-37 | 36.255 | 2.937 | Upregulated DNA damage pathway mutations |
| TYMS | 0.002 | 0.010 | 5.016 | 2.12E-04 | 2.20E-04 | 3.658 | 2.326 | Upregulated DNA damage pathway mutations |
| UBE2A | 0.002 | 0.019 | 7.797 | 5.08E-09 | 6.08E-09 | 8.216 | 2.963 | Upregulated DNA damage pathway mutations |
| UBE2B | 0.001 | 0.007 | 6.671 | 0.001 | 5.83E-04 | 3.234 | 2.738 | Upregulated DNA damage pathway mutations |
| UBE2C | 0.001 | 0.009 | 7.147 | 6.20E-05 | 6.48E-05 | 4.189 | 2.837 | Upregulated DNA damage pathway mutations |
| UBE2N | 0.002 | 0.011 | 6.988 | 1.08E-05 | 1.15E-05 | 4.939 | 2.805 | Upregulated DNA damage pathway mutations |
| UBE2S | 0.001 | 0.014 | 16.677 | 6.86E-10 | 8.69E-10 | 9.061 | 4.060 | Upregulated DNA damage pathway mutations |
| UBE2T | 0.002 | 0.007 | 3.924 | 0.006 | 0.006 | 2.248 | 1.972 | Upregulated DNA damage pathway mutations |
| UIMC1 | 0.004 | 0.038 | 8.815 | 2.42E-18 | 4.85E-18 | 17.314 | 3.140 | Upregulated DNA damage pathway mutations |
| USP1 | 0.005 | 0.041 | 8.286 | 4.43E-19 | 9.29E-19 | 18.032 | 3.051 | Upregulated DNA damage pathway mutations |
| USP10 | 0.005 | 0.061 | 11.245 | 3.11E-32 | 1.58E-31 | 30.801 | 3.491 | Upregulated DNA damage pathway mutations |
| USP2 | 0.006 | 0.048 | 7.970 | 2.78E-21 | 6.54E-21 | 20.184 | 2.995 | Upregulated DNA damage pathway mutations |
| USP4 | 0.007 | 0.055 | 7.652 | 9.05E-24 | 2.43E-23 | 22.614 | 2.936 | Upregulated DNA damage pathway mutations |
| UVRAG | 0.005 | 0.052 | 10.138 | 3.63E-26 | 1.13E-25 | 24.949 | 3.342 | Upregulated DNA damage pathway mutations |
| VCP | 0.006 | 0.041 | 6.687 | 8.54E-17 | 1.55E-16 | 15.810 | 2.741 | Upregulated DNA damage pathway mutations |
| VIM | 0.006 | 0.035 | 6.231 | 7.97E-14 | 1.18E-13 | 12.927 | 2.639 | Upregulated DNA damage pathway mutations |
| VRK1 | 0.003 | 0.037 | 14.294 | 3.28E-22 | 8.34E-22 | 21.079 | 3.837 | Upregulated DNA damage pathway mutations |
| VRK3 | 0.004 | 0.031 | 7.941 | 2.68E-14 | 4.13E-14 | 13.384 | 2.989 | Upregulated DNA damage pathway mutations |
| WEE1 | 0.004 | 0.030 | 8.128 | 4.82E-14 | 7.23E-14 | 13.141 | 3.023 | Upregulated DNA damage pathway mutations |
| WRN | 0.012 | 0.083 | 7.125 | 7.27E-34 | 4.60E-33 | 32.337 | 2.833 | Upregulated DNA damage pathway mutations |
| WWOX | 0.005 | 0.042 | 8.880 | 2.87E-20 | 6.44E-20 | 19.191 | 3.151 | Upregulated DNA damage pathway mutations |
| XPA | 0.001 | 0.021 | 14.661 | 5.11E-13 | 7.20E-13 | 12.142 | 3.874 | Upregulated DNA damage pathway mutations |
| XPC | 0.004 | 0.047 | 10.996 | 1.37E-24 | 3.78E-24 | 23.422 | 3.459 | Upregulated DNA damage pathway mutations |
| XRCC1 | 0.003 | 0.048 | 14.612 | 2.74E-28 | 1.00E-27 | 26.999 | 3.869 | Upregulated DNA damage pathway mutations |
| XRCC5 | 0.006 | 0.051 | 8.810 | 6.91E-24 | 1.88E-23 | 22.727 | 3.139 | Upregulated DNA damage pathway mutations |
| XRCC6 | 0.004 | 0.043 | 10.006 | 4.99E-22 | 1.23E-21 | 20.911 | 3.323 | Upregulated DNA damage pathway mutations |
| YWHAB | 0.002 | 0.013 | 7.743 | 7.54E-07 | 8.33E-07 | 6.079 | 2.953 | Upregulated DNA damage pathway mutations |
| YWHAE | 0.002 | 0.013 | 6.520 | 2.94E-06 | 3.21E-06 | 5.493 | 2.705 | Upregulated DNA damage pathway mutations |
| YWHAG | 0.002 | 0.014 | 7.848 | 2.51E-07 | 2.81E-07 | 6.551 | 2.972 | Upregulated DNA damage pathway mutations |
| YWHAH | 0.001 | 0.014 | 12.128 | 7.27E-09 | 8.64E-09 | 8.064 | 3.600 | Upregulated DNA damage pathway mutations |
| YWHAQ | 0.002 | 0.019 | 8.168 | 3.06E-09 | 3.68E-09 | 8.434 | 3.030 | Upregulated DNA damage pathway mutations |
| YWHAZ | 0.002 | 0.017 | 7.261 | 7.31E-08 | 8.33E-08 | 7.079 | 2.860 | Upregulated DNA damage pathway mutations |
| YY1 | 0.003 | 0.024 | 9.132 | 4.14E-12 | 5.60E-12 | 11.252 | 3.191 | Upregulated DNA damage pathway mutations |
| ZC3HC1 | 0.005 | 0.032 | 6.870 | 1.55E-13 | 2.28E-13 | 12.642 | 2.780 | Upregulated DNA damage pathway mutations |
| Abbreviation: Freq, Frequency of DNA damage pathway genes mutation; W, mTOR pathway wild-type patients; M, mTOR pathway mutant-type patients; P, P value; P Adj, Bonferroni-adjusted P value. | | | | | | | | |

| **Table S7:** Pathway enriched in the comparison of mutant-type versus wild-type patients for the 8-gene signature involved in mTOR pathway in TCGA cancer tissues | | | | | | | |
| --- | --- | --- | --- | --- | --- | --- | --- |
| ID | Type | Size | ES | NES | *P* | *P* Adj | FDR q |
| CYTOKINE CYTOKINE RECEPTOR INTERACTION | Immunity | 203 | 0.516 | 1.816 | 1.07E-08 | 1.04E-06 | 8.85E-07 |
| NATURAL KILLER CELL MEDIATED CYTOTOXICITY | Immunity | 105 | 0.647 | 2.171 | 1.16E-08 | 1.04E-06 | 8.85E-07 |
| GRAFT VERSUS HOST DISEASE | Immunity | 17 | 0.909 | 2.168 | 6.54E-08 | 3.90E-06 | 3.33E-06 |
| CALCIUM SIGNALING PATHWAY | Cell Metabolism | 147 | -0.656 | -1.684 | 1.09E-06 | 4.86E-05 | 4.14E-05 |
| CELL CYCLE | DNA repair/replication | 118 | 0.547 | 1.872 | 4.21E-06 | 1.51E-04 | 1.28E-04 |
| T CELL RECEPTOR SIGNALING PATHWAY | Immunity | 98 | 0.551 | 1.838 | 2.31E-05 | 6.90E-04 | 5.88E-04 |
| ANTIGEN PROCESSING AND PRESENTATION | Immunity | 46 | 0.677 | 2.022 | 2.96E-05 | 7.58E-04 | 6.46E-04 |
| NEUROACTIVE LIGAND RECEPTOR INTERACTION | Other | 155 | -0.617 | -1.592 | 3.53E-05 | 7.91E-04 | 6.74E-04 |
| CYTOSOLIC DNA SENSING PATHWAY | Immunity | 40 | 0.691 | 1.981 | 9.76E-05 | 0.002 | 0.002 |
| P53 SIGNALING PATHWAY | Other | 63 | 0.567 | 1.771 | 4.97E-04 | 0.009 | 0.008 |
| DNA REPLICATION | DNA repair/replication | 36 | 0.655 | 1.841 | 9.93E-04 | 0.015 | 0.013 |
| HOMOLOGOUS RECOMBINATION | DNA repair/replication | 24 | 0.710 | 1.827 | 0.001 | 0.017 | 0.014 |
| NOD LIKE RECEPTOR SIGNALING PATHWAY | Immunity | 55 | 0.563 | 1.730 | 0.001 | 0.017 | 0.014 |
| INTESTINAL IMMUNE NETWORK FOR IGA PRODUCTION | Immunity | 29 | 0.663 | 1.761 | 0.002 | 0.026 | 0.023 |
| RIBOSOME | Protein folding | 84 | 0.483 | 1.587 | 0.002 | 0.026 | 0.023 |
| PROXIMAL TUBULE BICARBONATE RECLAMATION | Cell Metabolism | 21 | -0.771 | -1.601 | 0.003 | 0.032 | 0.028 |
| JAK STAT SIGNALING PATHWAY | Transcription | 111 | 0.439 | 1.492 | 0.003 | 0.032 | 0.028 |
| GLYCINE SERINE AND THREONINE METABOLISM | Cell Metabolism | 29 | -0.729 | -1.612 | 0.003 | 0.032 | 0.028 |
| PROTEASOME | Protein folding | 41 | 0.574 | 1.665 | 0.004 | 0.040 | 0.034 |
| E2F TARGETS | Transcription | 187 | 0.695 | 2.530 | 1.00E-10 | 1.67E-09 | 1.12E-09 |
| G2M CHECKPOINT | DNA repair/replication | 183 | 0.701 | 2.549 | 1.00E-10 | 1.67E-09 | 1.12E-09 |
| INTERFERON GAMMA RESPONSE | Immunity | 189 | 0.587 | 2.133 | 1.00E-10 | 1.67E-09 | 1.12E-09 |
| MYC TARGETS V1 | Transcription | 188 | 0.562 | 2.047 | 1.60E-10 | 2.00E-09 | 1.35E-09 |
| ALLOGRAFT REJECTION | Immunity | 176 | 0.572 | 2.060 | 2.62E-10 | 2.62E-09 | 1.76E-09 |
| INTERFERON ALPHA RESPONSE | Immunity | 91 | 0.640 | 2.105 | 1.01E-07 | 7.89E-07 | 5.31E-07 |
| MTORC1 SIGNALING | MTOR | 192 | 0.503 | 1.816 | 1.10E-07 | 7.89E-07 | 5.31E-07 |
| TNFA SIGNALING VIA NFKB | Immunity | 195 | 0.498 | 1.834 | 1.67E-07 | 1.04E-06 | 7.02E-07 |
| MITOTIC SPINDLE | DNA repair/replication | 196 | 0.502 | 1.851 | 2.90E-07 | 1.61E-06 | 1.08E-06 |
| INFLAMMATORY RESPONSE | Immunity | 183 | 0.480 | 1.746 | 2.24E-06 | 1.12E-05 | 7.54E-06 |
| IL6 JAK STAT3 SIGNALING | Immunity | 80 | 0.618 | 1.960 | 5.76E-06 | 2.62E-05 | 1.76E-05 |
| ESTROGEN RESPONSE LATE | Other | 173 | 0.472 | 1.693 | 7.87E-06 | 3.28E-05 | 2.21E-05 |
| MYC TARGETS V2 | Transcription | 58 | 0.655 | 2.047 | 8.69E-06 | 3.34E-05 | 2.25E-05 |
| MYOGENESIS | Other | 170 | -0.589 | -1.527 | 1.21E-04 | 4.31E-04 | 2.91E-04 |
| GLYCOLYSIS | Cell Metabolism | 190 | 0.419 | 1.522 | 4.50E-04 | 0.001 | 0.001 |
| UNFOLDED PROTEIN RESPONSE | Protein folding | 105 | 0.455 | 1.514 | 0.002 | 0.007 | 0.005 |
| Abbreviation: ES, enrichment score; NES, normalized enrichment score; *P*, P value; *P* Adj, adjusted P value; FDR, false discovery rate; FDR q, FDR-adjusted q value | | | | | | | |

| **Table S8:** Pathway enriched in the comparison of mutant-type versus wild-type patients for the 8-gene signature involved in mTOR pathway in IMvigor210 study | | | | | | | |
| --- | --- | --- | --- | --- | --- | --- | --- |
| ID | Type | Size | ES | NES | *P* | *P* Adj | FDR q |
| COMPLEMENT AND COAGULATION CASCADES | Immunity | 69 | 0.822 | 1.890 | 1.33E-08 | 2.44E-06 | 1.99E-06 |
| CYTOKINE CYTOKINE RECEPTOR INTERACTION | Immunity | 227 | 0.672 | 1.641 | 7.85E-08 | 7.18E-06 | 5.87E-06 |
| NEUROACTIVE LIGAND RECEPTOR INTERACTION | Other | 223 | 0.641 | 1.566 | 3.79E-06 | 2.31E-04 | 1.89E-04 |
| SYSTEMIC LUPUS ERYTHEMATOSUS | Immunity | 57 | 0.779 | 1.767 | 1.09E-05 | 4.97E-04 | 4.06E-04 |
| HEMATOPOIETIC CELL LINEAGE | Other | 79 | 0.730 | 1.696 | 3.89E-05 | 0.001 | 0.001 |
| INTESTINAL IMMUNE NETWORK FOR IGA PRODUCTION | Immunity | 41 | 0.800 | 1.768 | 8.36E-05 | 0.003 | 0.002 |
| PPAR SIGNALING PATHWAY | Cell Metabolism | 66 | 0.730 | 1.680 | 1.11E-04 | 0.003 | 0.002 |
| JAK STAT SIGNALING PATHWAY | Transcription | 127 | 0.653 | 1.571 | 3.02E-04 | 0.007 | 0.006 |
| CELL ADHESION MOLECULES CAMS | Other | 126 | 0.646 | 1.552 | 4.11E-04 | 0.008 | 0.007 |
| PRIMARY IMMUNODEFICIENCY | Immunity | 34 | 0.786 | 1.684 | 6.01E-04 | 0.011 | 0.009 |
| GRAFT VERSUS HOST DISEASE | Immunity | 38 | 0.759 | 1.661 | 9.32E-04 | 0.014 | 0.012 |
| AUTOIMMUNE THYROID DISEASE | Immunity | 38 | 0.759 | 1.660 | 9.43E-04 | 0.014 | 0.012 |
| LEISHMANIA INFECTION | Immunity | 71 | 0.682 | 1.579 | 0.001 | 0.018 | 0.015 |
| TYPE I DIABETES MELLITUS | Cell Metabolism | 42 | 0.741 | 1.639 | 0.001 | 0.018 | 0.015 |
| CHEMOKINE SIGNALING PATHWAY | Immunity | 176 | 0.593 | 1.442 | 0.002 | 0.018 | 0.015 |
| PRION DISEASES | Immunity | 34 | 0.767 | 1.642 | 0.002 | 0.018 | 0.015 |
| TYROSINE METABOLISM | Cell Metabolism | 39 | 0.737 | 1.620 | 0.002 | 0.025 | 0.020 |
| PRIMARY BILE ACID BIOSYNTHESIS | Cell Metabolism | 15 | 0.839 | 1.618 | 0.003 | 0.030 | 0.025 |
| T CELL RECEPTOR SIGNALING PATHWAY | Immunity | 104 | 0.631 | 1.501 | 0.003 | 0.031 | 0.025 |
| GLYCOLYSIS GLUCONEOGENESIS | Cell Metabolism | 56 | 0.686 | 1.550 | 0.003 | 0.031 | 0.025 |
| ASTHMA | Other | 23 | 0.791 | 1.615 | 0.004 | 0.031 | 0.025 |
| ALLOGRAFT REJECTION | Immunity | 35 | 0.742 | 1.598 | 0.004 | 0.031 | 0.025 |
| TOLL LIKE RECEPTOR SIGNALING PATHWAY | Immunity | 89 | 0.632 | 1.483 | 0.005 | 0.042 | 0.035 |
| CYTOSOLIC DNA SENSING PATHWAY | Other | 42 | 0.705 | 1.561 | 0.006 | 0.044 | 0.036 |
| RETINOL METABOLISM | Cell Metabolism | 58 | 0.663 | 1.509 | 0.006 | 0.044 | 0.036 |
| ANTIGEN PROCESSING AND PRESENTATION | Immunity | 70 | 0.653 | 1.510 | 0.006 | 0.044 | 0.036 |
| ALLOGRAFT REJECTION | Immunity | 189 | 0.715 | 1.717 | 4.12E-10 | 1.12E-08 | 8.25E-09 |
| COAGULATION | Other | 135 | 0.756 | 1.798 | 4.48E-10 | 1.12E-08 | 8.25E-09 |
| XENOBIOTIC METABOLISM | Cell Metabolism | 193 | 0.655 | 1.573 | 4.72E-06 | 7.86E-05 | 5.79E-05 |
| COMPLEMENT | Immunity | 198 | 0.649 | 1.561 | 1.01E-05 | 1.26E-04 | 9.26E-05 |
| INTERFERON GAMMA RESPONSE | Immunity | 198 | 0.638 | 1.535 | 2.87E-05 | 2.87E-04 | 2.11E-04 |
| PANCREAS BETA CELLS | Cell Metabolism | 34 | 0.802 | 1.721 | 2.38E-04 | 0.002 | 0.001 |
| INFLAMMATORY RESPONSE | Immunity | 197 | 0.601 | 1.445 | 8.39E-04 | 0.006 | 0.004 |
| BILE ACID METABOLISM | Cell Metabolism | 109 | 0.645 | 1.516 | 0.001 | 0.008 | 0.006 |
| MYC TARGETS V2 | Transcription | 58 | -0.518 | -1.635 | 0.003 | 0.018 | 0.013 |
| IL6 JAK STAT3 SIGNALING | Immunity | 86 | 0.652 | 1.521 | 0.004 | 0.018 | 0.013 |
| G2M CHECKPOINT | DNA repair/replication | 189 | -0.345 | -1.292 | 0.006 | 0.027 | 0.020 |
| INTERFERON ALPHA RESPONSE | Immunity | 95 | 0.625 | 1.460 | 0.008 | 0.034 | 0.025 |
| E2F TARGETS | Transcription | 195 | -0.333 | -1.275 | 0.010 | 0.037 | 0.027 |
| Abbreviation: ES, enrichment score; NES, normalized enrichment score; *P*, P value; *P* Adj, adjusted P value; FDR, false discovery rate; FDR q, FDR-adjusted q value | | | | | | | |
